# Supplementary material for: High-Throughput Prediction of Exfoliable Non-van der Waals Materials from a Universal Potential
Source: Nat Commun. 2026 Aug 25;17:8977. doi: 10.1038/s41467-026-76806-8 (PMC13507353; doi:10.1038/s41467-026-76806-8)
Supplement: Supplementary file 1 [file 41467_2026_76806_MOESM1_ESM.pdf]

High-Throughput Prediction of Exfoliable Non-van der Waals Materials from a Universal Potential
Supplementary Information

Tom Barnowsky [1,2] Carsten Timm [3,4] and Rico Friedrich [1,2,*]

[1] *Theoretical Chemistry, Technische Universität Dresden, 01062 Dresden, Germany*
[2] *Institute of Ion Beam Physics and Materials Research, Helmholtz-Zentrum Dresden-Rossendorf, 01328 Dresden, Germany*
[3] *Institute of Theoretical Physics, Technische Universität Dresden, 01062 Dresden, Germany*
[4] *Würzburg-Dresden Cluster of Excellence ctd.qmat, Technische Universität Dresden, 01062 Dresden, Germany*

(Dated: August 6, 2026)

I. SUPPLEMENTARY METHODS

A. The AFLOW Standard

The parameters employed in the Vienna Ab-initio Simulation Package (VASP) [1–5] were chosen according to the AFLOW standard [6]. This includes the selection of pseudopotentials (PPs) as listed in Supplementary Table 2, as well as on-site interaction parameters applied to selected d- and f-shell elements. Specifically, effective Hubbard U parameters are used for d-elements, while both U and J parameters are employed for f-elements [7–9], as detailed in Supplementary Tables 3 and 4.

Each PP provides a recommended minimum plane-wave cutoff energy (ENMAX). To ensure reliable convergence, the cutoff energy in each calculation is set to 1.4 times the maximum ENMAX among the PPs used.

AFLOW uses an automatic approach to generate $\vec{k}$-point grids for bulk material, "$\vec{k}$-points per reciprocal atom" (KPPRA). For the in-plane reciprocal space integration grids ($N_1 \times N_2 \times 1$) used for 2D cells a modified version was used. The parameter 2D-KPPRA is defined such that 2D-KPPRA $\leq N_{\mathrm{atoms}} N_1 N_2$, where N_{atoms} is the number of atoms in the unit cell. The distribution of sampling points N_i along the two in-plane reciprocal lattice vectors $\vec{b}_i$ is chosen to best match the reciprocal lattice geometry, *i.e.*, to approximately satisfy $||\vec{b}_1||/N_1 = ||\vec{b}_2||/N_2$.

II. SUPPLEMENTARY FIGURES

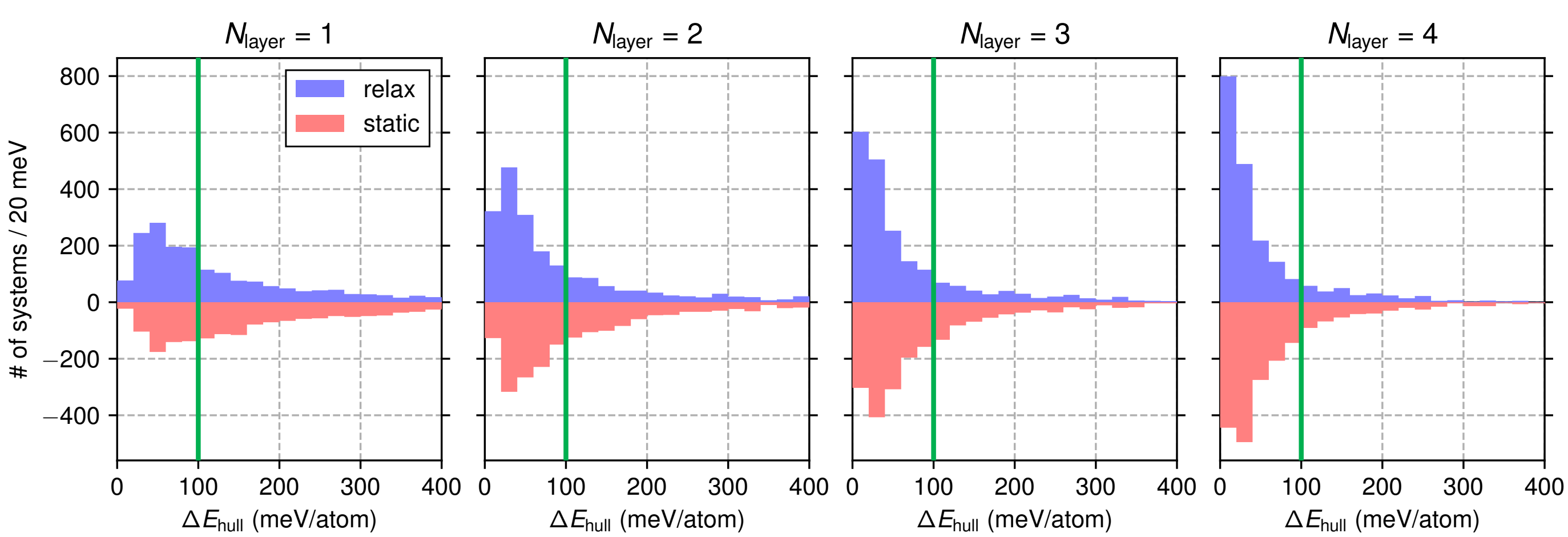

SUPPLEMENTARY FIGURE 1. **Layer dependence of the convex hull distance energy.** Distribution of convex hull distance energies ΔE_{hull} for the candidates as a function of the number of layers N_{layers}. "Relax" and "static" refer to calculations including and excluding structural relaxation of the derived 2D sheets, respectively. The common stability criterion $\Delta E_{\mathrm{hull}} = 100\,\mathrm{meV/atom}$ is indicated by the green vertical line.

* Corresponding author, e-mail: rico.friedrich@tu-dresden.de

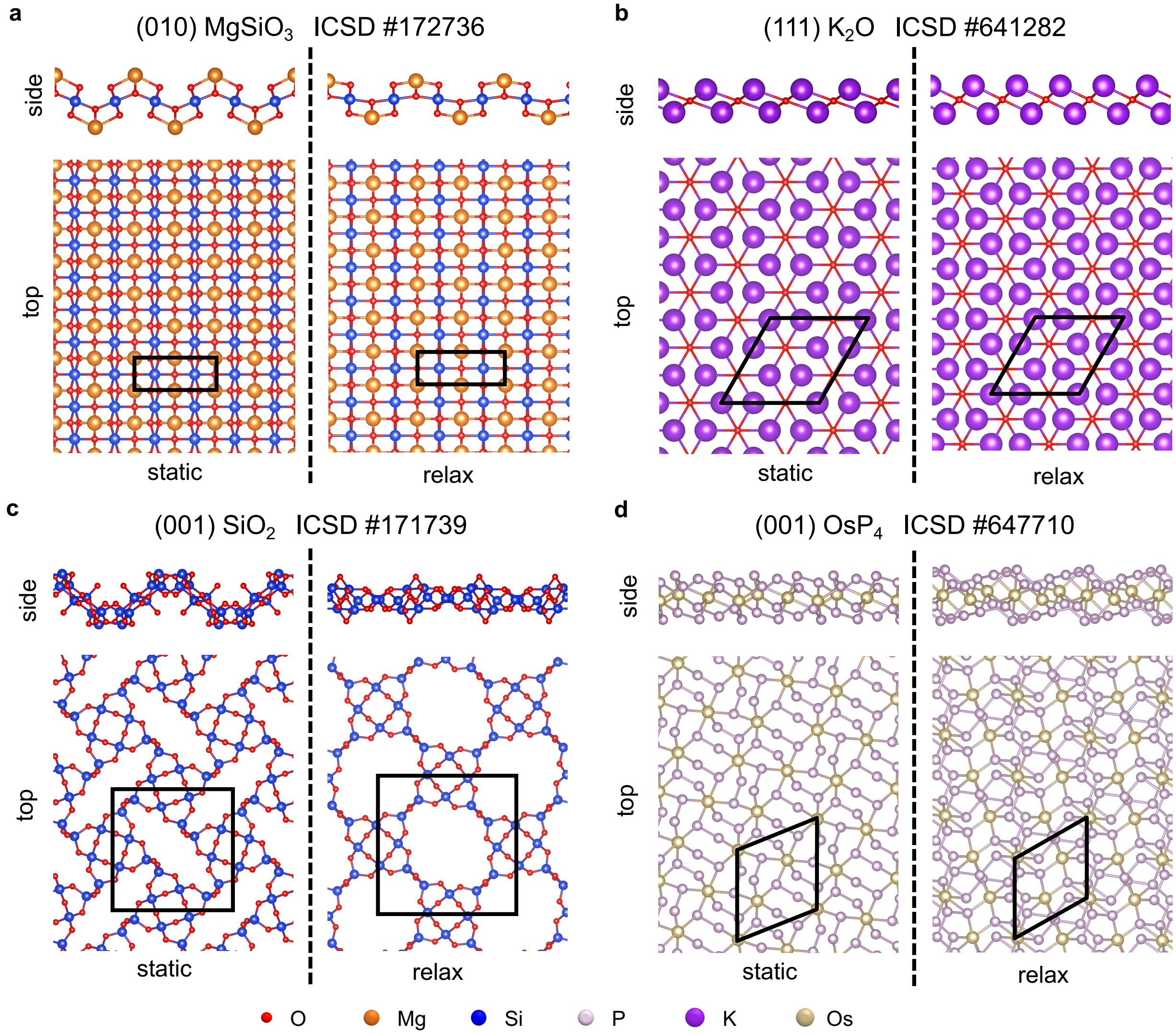

SUPPLEMENTARY FIGURE 2. **Examples for Relaxation Effects.** Top and side views comparing 2D structures immediately after cleavage ("static") and after structural optimization ("relax"). For each material, all views are displayed on a common scale to highlight relaxation-induced changes in area and thickness. Black frames indicate the in-plane unit cells.

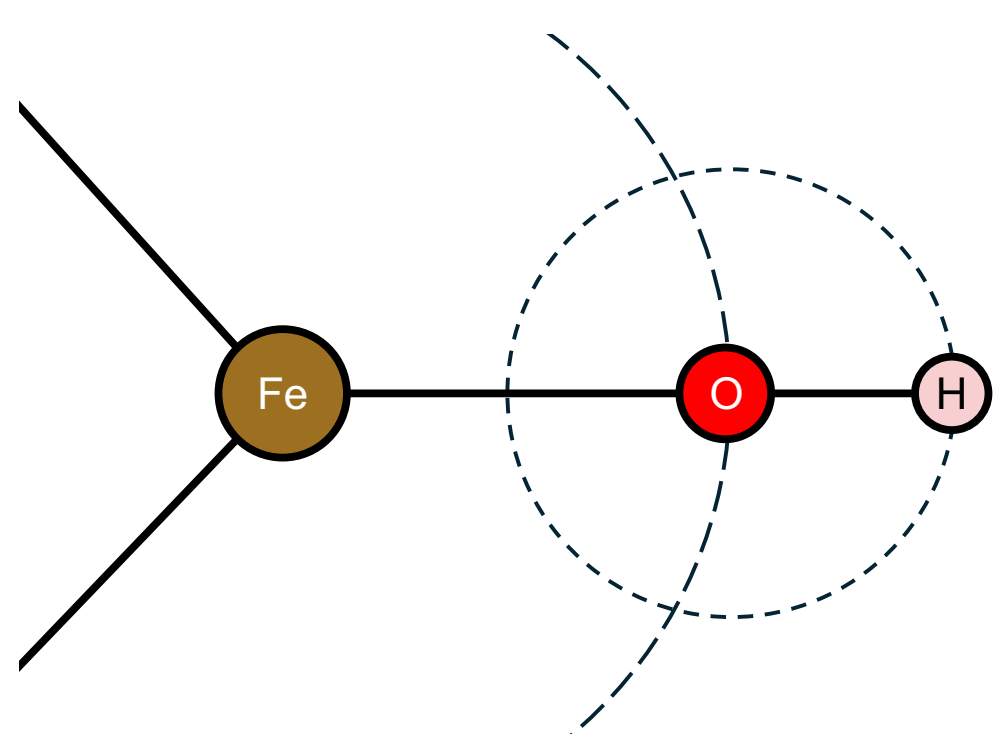

SUPPLEMENTARY FIGURE 3. **Asymmetry of Nearest Neighbor Radii.** Schematic visualization of a hypothetical iron-hydroxide bonding arrangement. Atoms are depicted as circles, nearest neighbor bonds as black lines. The nearest neighbor radius of the oxygen atom (small dashed circle) does not encompass iron, while the iron nearest neighbor radius (large dashed circle) encompasses oxygen.

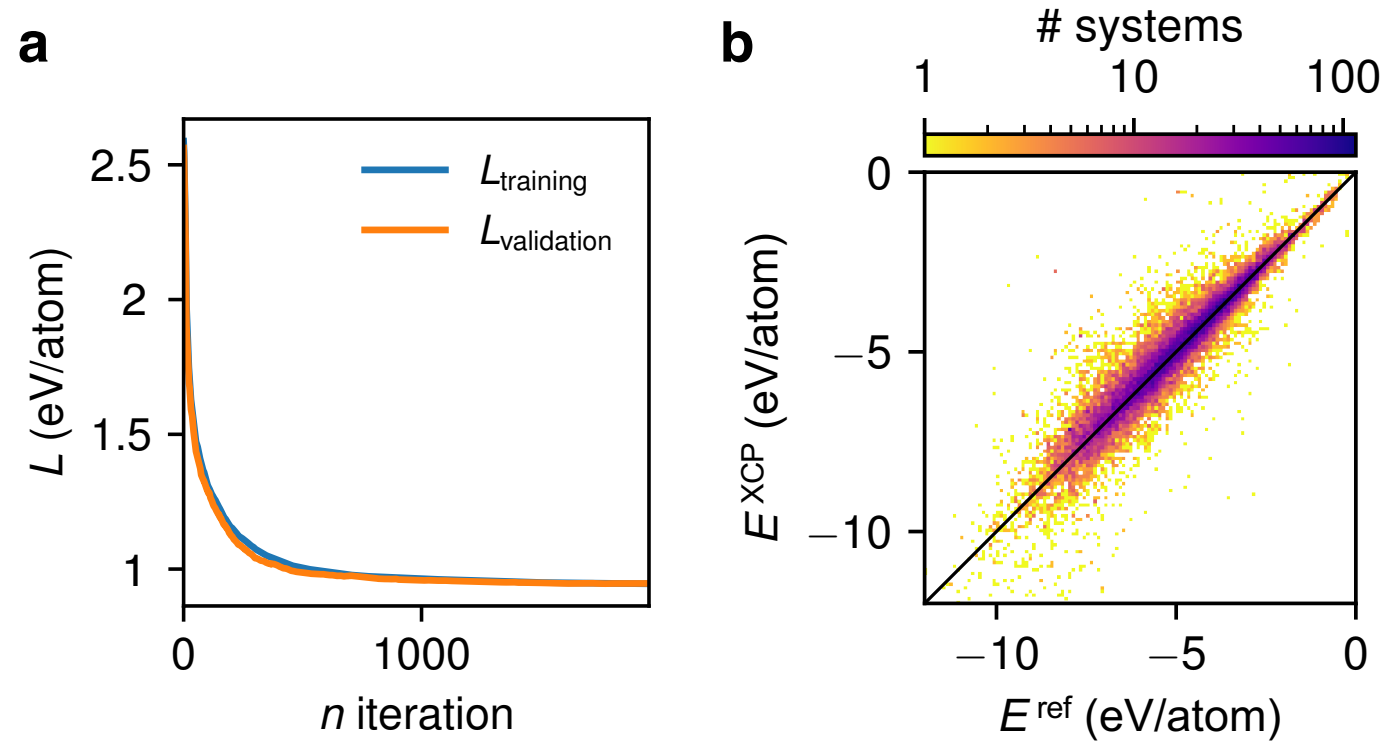

SUPPLEMENTARY FIGURE 4. **Loss Minimization.** (**a**) Training and validation loss during potential optimization. (**b**) Reference versus model energies for the final potential.

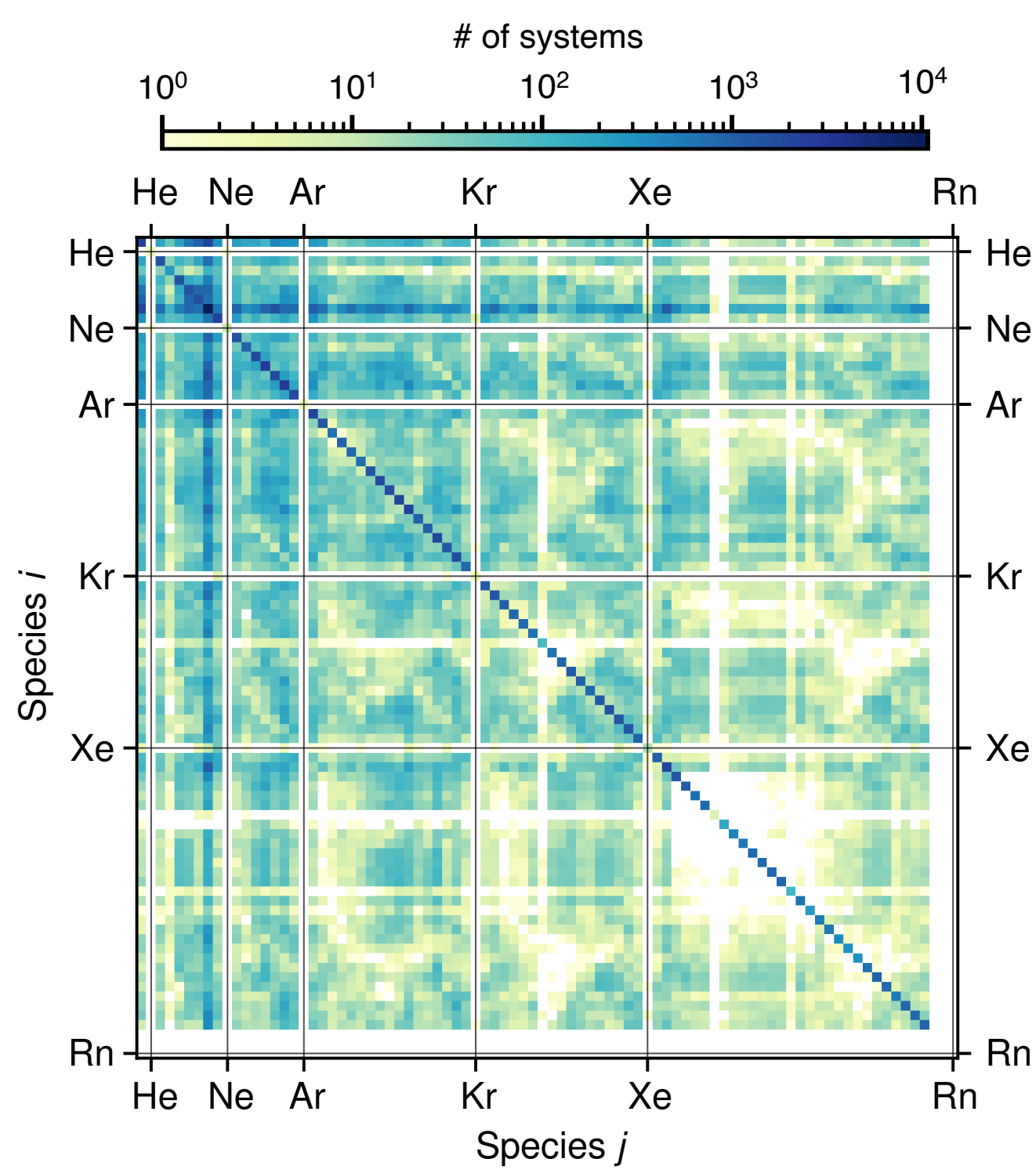

SUPPLEMENTARY FIGURE 5. **Representation Bias.** Distribution of bond pairs in the AFLOW-ICSD dataset. Each square in the matrix corresponds to a species pair (i, j), with the color indicating the number of systems exhibiting this interaction according to the neighbor determination criterion developed in this work. Systems containing multiple bond types are counted for each pair, *i.e.*, the total count exceeds the number of distinct systems.

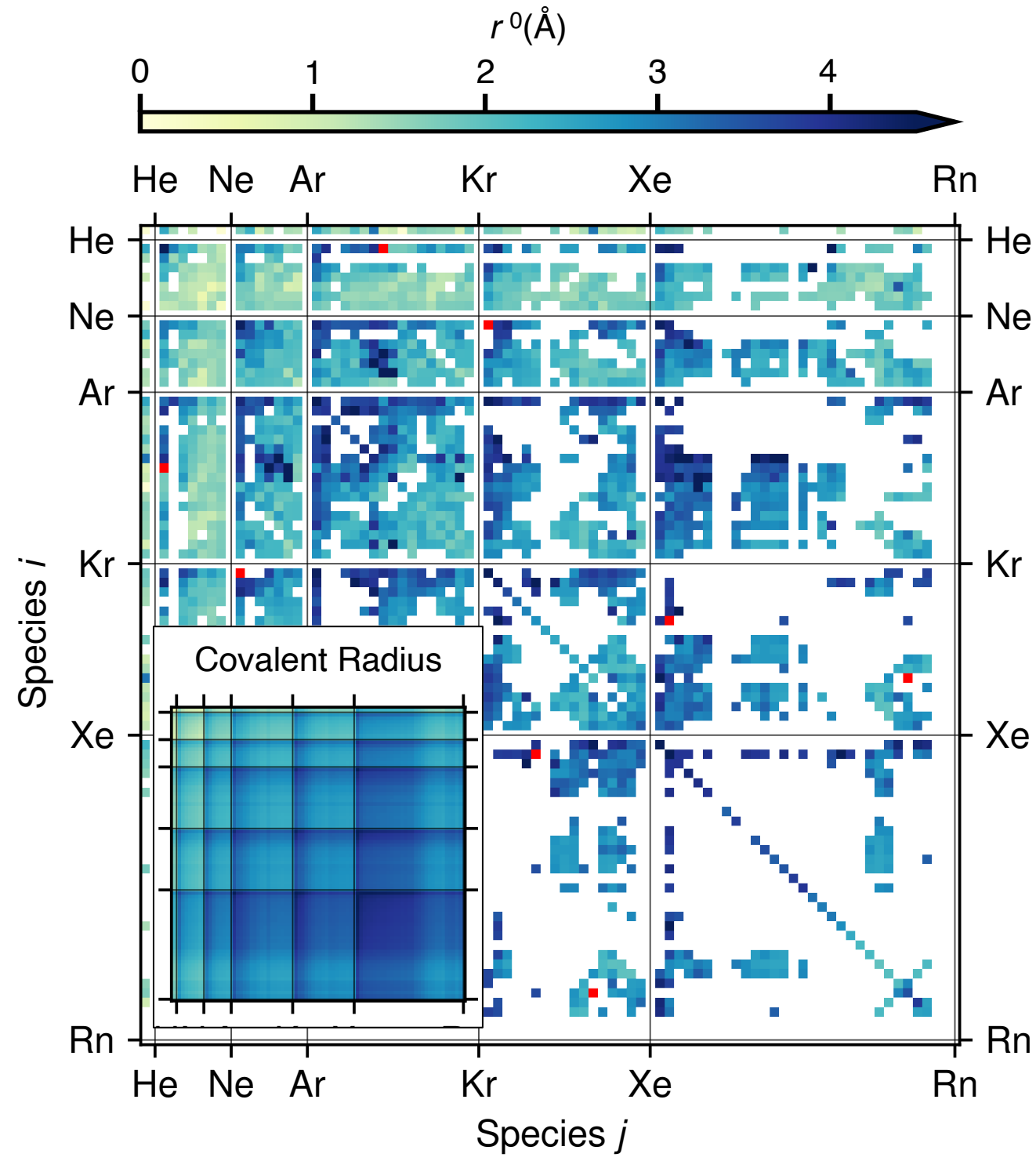

SUPPLEMENTARY FIGURE 6. **Equilibrium Radii.** Visualization of the numerically determined equilibrium radius of the fitted two-body potential, *i.e.*, $\operatorname{argmin} V(r)$. Each square in the matrix corresponds to a species pair (i, j), with the color scheme indicating the equilibrium bonding radii according to the adjacent bar. Missing data is shown in white; red squares mark pairs with no equilibrium radius. Data is symmetric about the diagonal as bonding is reciprocal. The inset shows the sum of covalent radii from Ref. [10].

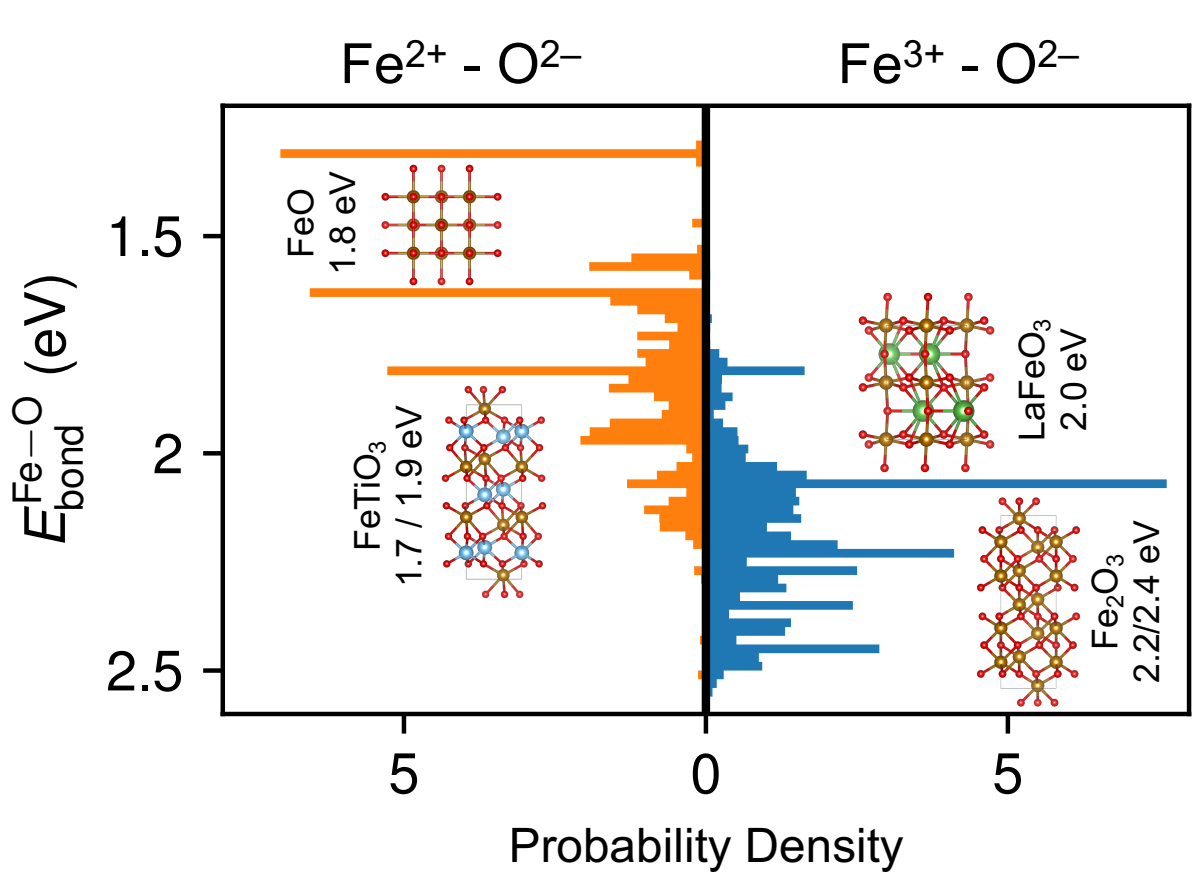

SUPPLEMENTARY FIGURE 7. **Different Chemical Environments.** Probability density of Fe–O bonding energies $E_{\mathrm{bond}}^{\mathrm{Fe-O}}$ in the AFLOW-ICSD dataset for Fe in the +2 and +3 oxidation states. As an inset a range of representative structures for the two oxidation states together with their Fe–O bonding energies are displayed. If a structure has two different bonding energies, *e.g.*, for short and long bonds, all energies are shown. Oxidation states are determined on a database scale using the approaches developed for the CCE framework [11].

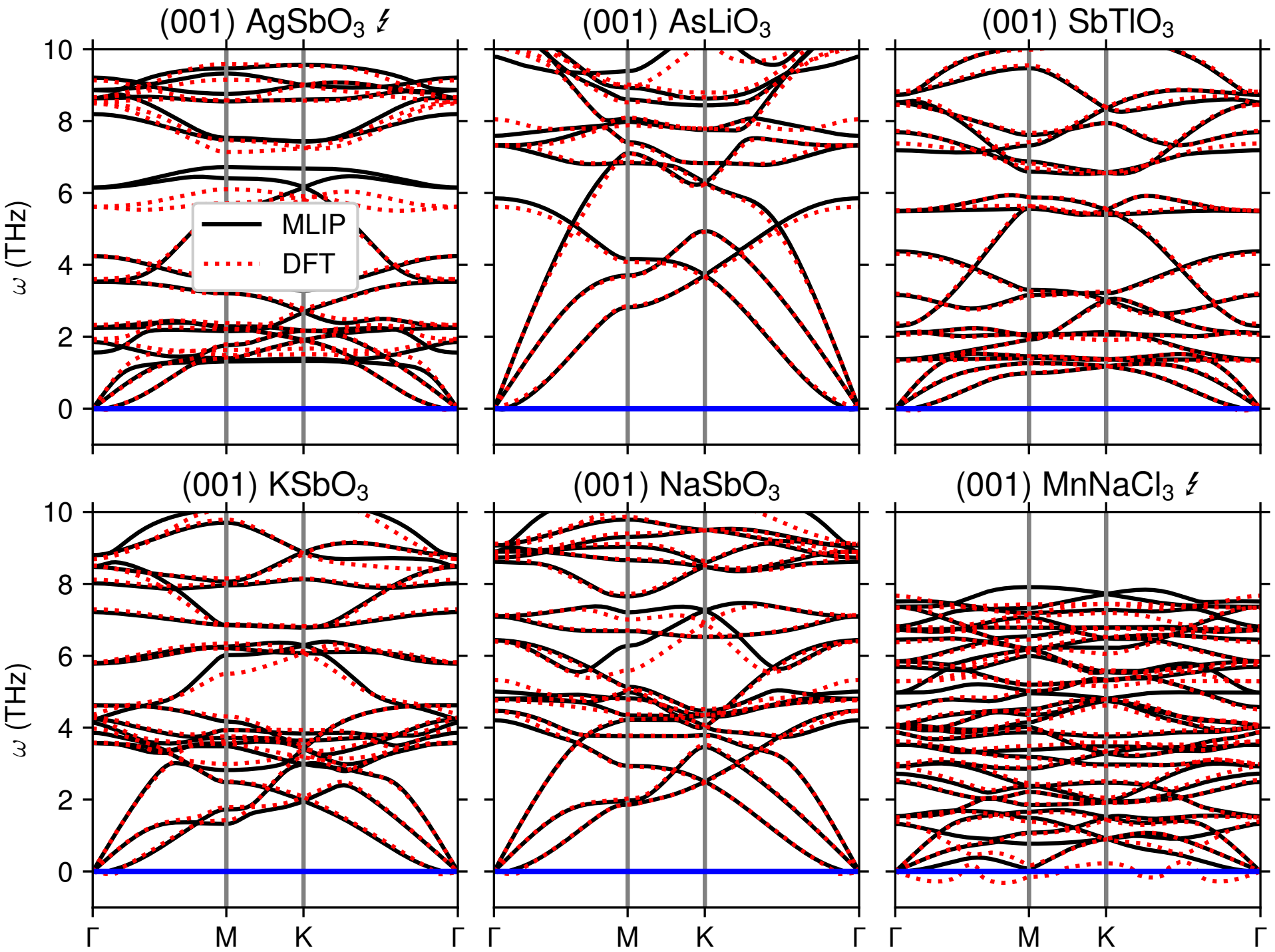

SUPPLEMENTARY FIGURE 8. **Phonon Calculation Benchmark.** *Ab-initio* phonon dispersions for non-vdW 2D candidates obtained from Ref. [12] (shown in red) in comparison to phonon dispersions determined using the mace-mh-1 MLIP (shown in black). Materials for which there is a large mismatch ($\epsilon > 0.1$) between the DFT and MLIP ground state structures are labeled with a lightning icon.

III. SUPPLEMENTARY TABLES

SUPPLEMENTARY TABLE 1. **Benchmark of the XCP Method with Respect to Experimentally Exfoliated Systems.** List of experimentally exfoliated non-vdW 2D materials, limited to studies where both the exfoliated plane and structure are explicitly stated in the original publication. The table reports the bulk compound, Miller indices of the exfoliated lattice plane, exfoliation technique (L = liquid-phase, M = mechanical), literature reference, as well as the bulk structure's ICSD database identifier, space group, and Pearson symbol. Entries are sorted by publication date. The last two columns indicate whether the exfoliated planes were correctly predicted by the HKLSEARCH (H) and BONDDEL (B) approaches. For successful matches, the corresponding exfoliability ratio is listed. The final row summarizes the overall success rate if both approaches are combined.

Material	Plane	Technique	Ref.	Bulk # ICSD	Bulk Space Group	Bulk Pearson Symbol	R_H	R_B
α-WO_3	(001)	L	[13]	27961	$P4/nmm$ (#129)	tP8	1.15	1.34
$LiMn_2O_4$	(111)	L	[14]	53603	$Fd\bar{3}m$ (#227)	cF56	1.15	
$LiFePO_4$	(010)	L	[14]	260571	$Pnma$ (#62)	oP28		
β-B	(104)	L	[15]	240997	$R\bar{3}m$ (#166)	hR105	1.09	
α-Fe_2O_3	(001)	L	[16]	161292	$R\bar{3}c$ (#167)	hR10		1.06
$FeTiO_3$	(001)	L	[17]	247547	$R\bar{3}$ (#148)	hR10		1.63
$FeCr_2O_4$	(111)	L	[18]	183962	$Fd\bar{3}m$ (#227)	cF56	1.11	
MnTe	(110)	L	[19]	174028	$P6_3/mmc$ (#194)	hP4	1.02	
Si	(111)	L	[20]	652255	$Fd\bar{3}m$ (#227)	cF8	1.13	
Mg	(001)	L	[21]	642653	$P6_3/mmc$ (#194)	hP2	1.02	1.02
α-Ti	(001)	L	[22]	44872	$P6_3/mmc$ (#194)	hP2	1.01	1.05
PbS	(001)	L	[23]	169770	$Fm\bar{3}m$ (#225)	cF8	1.00	
$CaCO_3$	(104)	L	[23]	79673	$R\bar{3}c$ (#167)	hR10	1.82	
α-Ge	(111)	L	[24]	44841	$Fd\bar{3}m$ (#227)	cF8	1.13	
Fe_3O_4	(111)	L	[25]	249047	$Fd\bar{3}m$ (#227)	cF56	1.05	
B_4C	(100)	L, M	[26]	654971	$R\bar{3}m$ (#166)	hR15		
Sn	(111)	L	[27]	53789	$Fd\bar{3}m$ (#227)	cF8	1.13	
TiC	(110)	L	[28]	618935	$Fm\bar{3}m$ (#225)	cF8		
α-B	(101)	L	[29]	26487	$R\bar{3}m$ (#166)	hR12		
$AgCrS_2$	(001)	L	[30]	42396	$R3m$ (#160)	hR4	3.18	1.25
$InGaS_3$	(001)	M	[31]	62929	$P6_1$ (#169)	hP30		
Bi	(001)	M	[32]	616519	$R\bar{3}m$ (#166)	hP2		1.26
Sb	(001)	M	[32]	651490	$R\bar{3}m$ (#166)	hP2		1.18
SnO	(001)	M	[32]	16481	$P4/nmm$ (#129)	tP4	2.45	5.85
V_2O_5	(010)	M	[32]	41030	$Pmmn$ (#59)	oP14	3.17	4.67
Bi_2O_2Se	(001)	M	[32]	411143	$I4/mmm$ (#139)	tI10	1.45	3.42
$CaMn_4Si_5O_{15}$	(110)	L	[33]	200162	$P\bar{1}$ (#2)	aP50		
CaF_2	(111)	L	[34]	44937	$Fm\bar{3}m$ (#225)	cF12	1.16	
Cr_2S_3	(001)	L	[35]	16720	$P\bar{3}1c$ (#163)	hP20	1.29	
$\sum =$							23/29	

SUPPLEMENTARY TABLE 2. **Pseudopotential Choice.** Employed VASP PPs for the PBE functional according to the AFLOW standard. Each element is associated with a specific PP which is labeled in accordance with the VASP convention.

Element	Label	Element	Label	Element	Label	Element	Label	Element	Label
Ac	Ac	Cs	Cs_sv	K	K_sv	Pa	Pa	Sr	Sr_sv
Ag	Ag	Cu	Cu_pv	Kr	Kr	Pb	Pb_d	Ta	Ta_pv
Al	Al	Dy	Dy_3	La	La	Pd	Pd_pv	Tb	Tb_3
Ar	Ar	Er	Er_3	Li	Li_sv	Pm	Pm	Tc	Tc_pv
As	As	Eu	Eu	Lu	Lu	Pt	Pt	Te	Te
Au	Au	F	F	Mg	Mg_pv	Pr	Pr	Th	Th_s
B	B_h	Fe	Fe_pv	Mn	Mn_pv	Pu	Pu_s	Ti	Ti_sv
Ba	Ba_sv	Ga	Ga_h	Mo	Mo_pv	Rb	Rb_sv	Tl	Tl_d
Be	Be_sv	Ge	Ge_h	Na	Na_sv	Re	Re_pv	Tm	Tm
Bi	Bi_d	Gd	Gd	N	N	Rh	Rh_pv	U	U
Br	Br	H	H	Nb	Nb_sv	Ru	Ru_pv	V	V_sv
C	C	He	He	Nd	Nd	S	S	W	W_sv
Ca	Ca_sv	Hf	Hf_pv	Ne	Ne	Sb	Sb	Xe	Xe
Cd	Cd	Hg	Hg	Ni	Ni_pv	Sc	Sc_sv	Y	Y_sv
Ce	Ce	Ho	Ho_3	Np	Np_s	Se	Se	Yb	Yb
Cl	Cl	I	I	O	O	Si	Si	Zn	Zn
Co	Co	In	In_d	Os	Os_pv	Sm	Sm	Zr	Zr_sv
Cr	Cr_pv	Ir	Ir	P	P	Sn	Sn		

SUPPLEMENTARY TABLE 3. *d***-Element On-Site Parameters.** Effective Hubbard U parameters applied to d-shell elements.

Element	U_{eff}	Element	U_{eff}	Element	U_{eff}	Element	U_{eff}	Element	U_{eff}
Sc	2.9	Ni	5.1	Tc	2.7	Ta	2.0	Hg	5.1
Ti	4.4	Cu	4.0	Ru	3.0	W	2.2	Ga	3.9
V	2.7	Zn	7.5	Rh	3.3	Re	2.4	In	1.9
Cr	3.5	Y	5.1	Pd	3.6	Os	2.6	Bi	0.0
Mn	4.0	Zr	5.1	Ag	4.0	Ir	2.8	Pb	0.0
Fe	4.6	Nb	2.1	Cd	2.1	Pt	3.0	Sn	3.5
Co	5.0	Mo	2.4	Hf	5.1	Au	4.0		

SUPPLEMENTARY TABLE 4. *f***-Element On-Site Parameters.** U and J parameters applied to selected f-shell elements.

Element	U	J	Element	U	J
La	8.1	0.6	Tm	7.0	1.0
Ce	7.0	0.7	Dy	5.6	0.0
Pr	6.5	1.0	Yb	7.0	0.7
Gd	6.7	0.7	Lu	4.8	0.9
Nd	7.2	1.0	U	4.5	0.5
Sm	7.4	1.0	Th	5.0	0.0
Eu	6.4	1.0			

SUPPLEMENTARY REFERENCES

[1] Kresse, G. & Hafner, J. *Ab initio* molecular dynamics for liquid metals. *Phys. Rev. B* **47**, 558–561 (1993).
[2] Blöchl, P. E., Jepsen, O. & Andersen, O. K. Improved tetrahedron method for Brillouin-zone integrations. *Phys. Rev. B* **49**, 16223–16233 (1994).
[3] Kresse, G. & Hafner, J. Norm-Conserving and Ultrasoft Pseudopotentials for First-Row and Transition-Elements. *J. Phys.: Condens. Matter* **6**, 8245–8257 (1994).
[4] Kresse, G. & Furthmüller, J. Efficient iterative schemes for *ab initio* total-energy calculations using a plane-wave basis set. *Phys. Rev. B* **54**, 11169–11186 (1996).
[5] Kresse, G. & Furthmüller, J. Efficiency of *ab-initio* total energy calculations for metals and semiconductors using a plane-wave basis set. *Comput. Mater. Sci.* **6**, 15–50 (1996).
[6] Calderon, C. E. *et al.* The AFLOW standard for high-throughput materials science calculations. *Comput. Mater. Sci.* **108 Part A**, 233–238 (2015).
[7] Anisimov, V. I., Zaanen, J. & Andersen, O. K. Band theory and Mott insulators: Hubbard U instead of Stoner I. *Phys. Rev. B* **44**, 943–954 (1991).
[8] Liechtenstein, A. I., Anisimov, V. I. & Zaanen, J. Density-functional theory and strong interactions: Orbital ordering in Mott-Hubbard insulators. *Phys. Rev. B* **52**, R5467 (1995).
[9] Dudarev, S. L., Botton, G. A., Savrasov, S. Y., Humphreys, C. J. & Sutton, A. P. Electron-energy-loss spectra and the structural stability of nickel oxide: An LSDA+U study. *Phys. Rev. B* **57**, 1505–1509 (1998).
[10] Cordero, B. *et al.* Covalent radii revisited. *Dalton Trans.* **37**, 2832–2838 (2008).
[11] Friedrich, R. *et al.* Coordination corrected ab initio formation enthalpies. *npj Comput. Mater.* **5**, 59 (2019).
[12] Barnowsky, T., Krasheninnikov, A. V. & Friedrich, R. A New Group of 2D Non-van der Waals Materials with Ultra Low Exfoliation Energies. *Adv. Electron. Mater.* **9**, 2201112 (2023).
[13] Guan, G. *et al.* Electrostatic-Driven Exfoliation and Hybridization of 2D Nanomaterials. *Adv. Mater.* **29**, 1700326 (2017).
[14] Tai, Z. *et al.* Few Atomic Layered Lithium Cathode Materials to Achieve Ultrahigh Rate Capability in Lithium-Ion Batteries. *Adv. Mater.* **29**, 1700605 (2017).
[15] Li, H. *et al.* Scalable Production of Few-Layer Boron Sheets by Liquid-Phase Exfoliation and Their Superior Supercapacitive Performance. *ACS Nano* **12**, 1262–1272 (2018).
[16] Puthirath Balan, A. *et al.* Exfoliation of a non-van der Waals material from iron ore hematite. *Nat. Nanotechnol.* **13**, 602–609 (2018).
[17] Puthirath Balan, A. *et al.* A Non-van der Waals Two-Dimensional Material from Natural Titanium Mineral Ore Ilmenite. *Chem. Mater.* **30**, 5923–5931 (2018).
[18] Yadav, T. P. *et al.* Chromiteen: A New 2D Oxide Magnetic Material from Natural Ore. *Adv. Mater. Interfaces* **5**, 1800549 (2018).
[19] Puthirath Balan, A. *et al.* Magnetic Properties and Photocatalytic Applications of 2D Sheets of Nonlayered Manganese Telluride by Liquid Exfoliation. *ACS Appl. Nano Mater.* **1**, 6427–6434 (2018).
[20] Wang, Y. *et al.* Directly Exfoliated Ultrathin Silicon Nanosheets for Enhanced Photocatalytic Hydrogen Production. *J. Phys. Chem. Lett.* **11**, 8668–8674 (2020).
[21] Zhang, C. *et al.* Cryogenic Exfoliation of Non-layered Magnesium into Two-Dimensional Crystals. *Angew. Chem. Int. Ed.* **58**, 8814–8818 (2019).
[22] Xie, Z. *et al.* Biocompatible Two-Dimensional Titanium Nanosheets for Multimodal Imaging-Guided Cancer Theranostics. *ACS Appl. Mater. Interfaces* **11**, 22129–22140 (2019).
[23] Liu, S. *et al.* Facile preparation of novel and active 2D nanosheets from non-layered and traditionally non-exfoliable earth-abundant materials. *J. Mater. Chem. A* **7**, 15411–15419 (2019).
[24] Feng, C. *et al.* Germanene-Based Theranostic Materials for Surgical Adjuvant Treatment: Inhibiting Tumor Recurrence and Wound Infection. *Matter* **3**, 127–144 (2020).
[25] Puthirath, A. B. *et al.* Scale-Enhanced Magnetism in Exfoliated Atomically Thin Magnetite Sheets. *Small* **16**, 2004208 (2020).
[26] Guo, Y. *et al.* Exfoliation of boron carbide into ultrathin nanosheets. *Nanoscale* **13**, 1652–1662 (2021).
[27] Ouyang, J. *et al.* Cryogenic Exfoliation of 2D Stanene Nanosheets for Cancer Theranostics. *Nano-Micro Lett.* **13**, 90 (2021).
[28] Hu, C.-X. *et al.* Solvent-Assisted Anisotropic Cleavage of Transition Metal Carbide into 2D Nanoflakes. *Small Struct.* **2**, 2100039 (2021).
[29] Göktuna, S. & Taşaltın, N. Preparation and characterization of PANI: α borophene electrode for supercapacitors. *Physica E* **134**, 114833 (2021).
[30] Peng, J. *et al.* Stoichiometric two-dimensional non-van der Waals $AgCrS_2$ with superionic behaviour at room temperature. *Nat. Chem.* **13**, 1235–1240 (2021).
[31] Toksumakov, A. N. *et al.* High-refractive index and mechanically cleavable non-van der Waals $InGaS_3$. *npj 2D Materials and Applications* **6**, 85 (2022).
[32] Jiang, K. *et al.* Mechanical cleavage of non-van der Waals structures towards two-dimensional crystals. *Nat. Synth.* **2**, 58–66 (2023).
[33] Mahapatra, P. L. *et al.* Paramagnetic two-dimensional silicon-oxide from natural silicates. *2D Mater.* **11**, 015019 (2023).
[34] Chattopadhyay, S. *et al.* Unlocking the Potential: Atomically Thin 2D Fluoritene from Exfoliated Fluorite Ore and Its Electrochemical Activity. *Nano Lett.* **24**, 7284–7292 (2024).

[35] Su, W. *et al.* Liquid Phase Exfoliation of Few-Layer Non-Van der Waals Chromium Sulfide. *Adv. Sci.* **11**, 2402875 (2024).
