# Supplementary material for: High-Throughput Prediction of Exfoliable Non-van der Waals Materials from a Universal Potential
Source: Nat Commun. 2026 Aug 25;17:8977. doi: 10.1038/s41467-026-76806-8 (PMC13507353; doi:10.1038/s41467-026-76806-8)
Supplement: Supplementary file 2 [file 41467_2026_76806_MOESM2_ESM.pdf]

nature portfolio

Peer Review File

High-Throughput Prediction of Exfoliable Non-van der Waals Materials from a Universal Potential

Corresponding Author: Dr Rico Friedrich

This file contains all reviewer reports in order by version, followed by all author rebuttals in order by version.

Version 0:

Reviewer comments:

Reviewer #1

(Remarks to the Author)
Judging the current manuscript against its title, which emphasizes “exfoliation and cleavage of available bulk crystals,” the central idea has been explored in previous studies (such as npj Computational Materials, 7(1), 147): 17062). Although the methods are different and while this manuscript does offer advantages over earlier works, these advances, in my view, are not sufficient to warrant publication in Nature Communications. By contrast, the alternative framing of the work—namely, the identification of a new class of two-dimensional materials via high-throughput theoretical methods—is considerably more compelling. I agree with the authors that the search for non–van der Waals (non-vdW) monolayers has so far relied heavily on trial-and-error approaches, and that a systematic theoretical investigation is indeed needed. However, the present manuscript does not report computational results required to support that the proposed 2,377 monolayers are stable and experimentally meaningful 2D materials.

Based on the information provided in the manuscript and the data availability webpage, MD simulations appear to have been performed for only a very limited number of systems (specifically those shown in Figure 4). In the absence of further clarification, this suggests that the stability analysis is restricted to six systems, which is insufficient to build confidence in a dataset exceeding 2,000 proposed non-vdW monolayers. I consider a robust stability analysis essential before proposing a new class of stable 2D materials. The authors themselves note the high surface reactivity of non-vdW systems (line 38), which further reinforces this concern. While neither phonon calculations nor convex-hull analysis can fully guarantee experimental stability, they are widely accepted as minimum baseline criteria in the field. Had such analyses been performed in a systematic manner—particularly in combination with the existence of the parent bulk phases—I would have regarded this work as a meaningful step forward.

The authors largely base their claim regarding the existence of stable non–van der Waals (non-vdW) 2D materials on previously reported experimental cases. While such reports provide motivation for the present study, and while the results could potentially help guide future experimental efforts, several critical issues must be considered. Given that most experimental studies in the literature (as collected in the Supporting Information) rely on liquid-phase exfoliation, two fundamental theoretical concerns must be addressed when proposing the resulting products as candidate 2D materials:

1. Monolayer versus few-layer stability. Are these materials stable as true monolayers, or do they only stabilize as few-layer thin films?
2. Chemical stability upon exfoliation. Since exfoliation from the bulk necessarily involves breaking strong covalent or ionic bonds, are the resulting layers intrinsically stable, or are they instead stabilized by passivation from ions or molecules present in the liquid environment?

As the present work is purely computational, I do not expect the authors to resolve these experimentally driven concerns directly. However, these considerations indicate that the existing experimental reports alone are insufficient to establish the existence of a stable class of non-vdW 2D materials, and that any theoretical claim must therefore be supported by a systematic stability analysis.

For these reasons, I cannot recommend publication of this manuscript in Nature Communications at this stage. I would, however, be willing to reconsider my recommendation if the following major issues are addressed:

1. Stability analysis: Phonon band calculations are essential, and convex-hull analysis is strongly encouraged, for the proposed 2D materials.
2. Structural analysis: The authors have access to structural data before and after monolayer relaxation, yet no quantitative analysis of bond deformation or reconstruction is presented. This analysis is crucial, as the novelty of these candidate 2D materials arises precisely from the non-intuitive stability of layers formed by breaking strong non-vdW bonds. The authors are in a unique position to comment on how the structures adjust upon exfoliation.

Specific comments on the manuscript:

• Abstract: The authors state, “we obtain 37,208 cleavable surfaces … from which we investigate 2,377 likely exfoliable ones.” The criteria used to classify a surface as “cleavable” versus “exfoliable” must be explicitly defined.

• Line 76: The selection criteria for 2D material candidates are central to this work. Although Ref. [31] is cited, the relevant criteria should be clearly summarized in the present manuscript.

• Line 79: The authors refer to “properties” studied for the 2D candidates. Please specify for what fraction of the 2,377 monolayers MD and band-structure calculations were performed.

• Line 158: Please clarify the meaning of “non-planar cleavage.”

• Line 182: Wulff constructions can provide insight into whether a material prefers to form thin films or three-dimensional clusters. Can the authors present or discuss Wulff-shape analysis for the systems studied?

• Line 270: The authors state that exfoliation energies are calculated only for systems “whose composition is conserved.” Please clarify how this criterion is applied in practice. Specifically, how many (or what fraction) of the 2,377 proposed exfoliable monolayers preserve their composition and are therefore carried forward to the AFLOW/VASP calculations? Alternatively, does the number 2,377 already correspond to this subset? Please also clarify whether lattice relaxations are performed exclusively for these systems, and how many materials are excluded based on this criterion.

• Line 273: What is the origin of the 16,397 systems mentioned here? Why is this number smaller than the total number of 2D systems discussed earlier?

• Line 282: The surface termination analysis is interesting; however, given that liquid-phase exfoliation occurs in a chemically active and uncontrolled environment, do the authors expect that experimentally realized thin films may be stabilized by surface passivation?

• Line 359: How were the Hubbard U values chosen for each element and compound? Were these U values also used during structural relaxations? While such information may exist in previous AFLOW publications, I recommend that sufficient computational details be provided to make the present manuscript self-contained.

(Remarks on code availability)
I did not review the codes.

Reviewer #2

(Remarks to the Author)
Barnowsky et al. have explored non-van der Waals (non-vdW) 2D materials by computational exfoliation and cleavage of known crystals. They constructed a model potential (XCP) that is a sum of Morse and Yukawa potentials, which computes the bond strength with small computational costs. They also introduced BONDDEL and HKLSEARCH schemes, which predict known non-vdW 2D materials such as alpha-WO3 and silicene, respectively, and defined the exfoliability ratio R in Eq. (2). Using these approaches, they proposed that 2377 exfoliable monolayers from non-vdW materials. The statistical analysis and electronic structure calculations have also been performed. finding It remains an open question how to predict non-vdW 2D materials, so the idea proposed in the present manuscript is interesting. However, I do not agree that this approach successfully identifies known 2D materials and accurately predicts likely exfoliable ones.

Major comments
At line 280, the authors state that “we decided to perform a detailed high-throughput analysis on the 2377 systems with R>1.5”. On the other hand, only 6 out of 29 experimentally exfoliated non-vdW 2D materials satisfy the criteria of R>1.5, and even hematene (R=1.06) and silinece (R=1.13) do not satisfy the criteria, which are shown in Supporting Information. There is a clear inconsistency between the experimental results and the predictions, indicating an apparent contradiction in this manuscript.

In my understanding, the surface energy, corresponding to R_H estimated by the HKLSEARCH, may not be an adequate descriptor for predicting non-vdW 2D materials. As mentioned in lines 333-337, the final structures are determined by the surface energy. However, the growth process of non-vdW 2D materials may not be solely determined by the surface energy.

Minor comments
Lines 62-64. The discussion of Refs. [24-27] is rather superficial. A more careful discussion is needed to clarify the

differences from the present work.

Lines 396-398. The six systems in Fig. 4 were found to be dynamically stable through density functional MD simulations. Does this guarantee no unstable phonon mode in the Brillouin zone? Why were phonon dispersions not calculated?

(Remarks on code availability)
Example files for FINDSLAB_POTDATA are not provided, and I could not run the code.

Reviewer #3

(Remarks to the Author)
In this work the authors propose a general methodology to discover new two-dimensional materials starting from non-Van der Waals bonded bulk crystals and then they apply such methodology to a large database of pre-processed crystal structures (i.e. structure coming from an experimental database but previously optimized with density functional theory). In their quest the authors successfully identified a large number of possible two-dimensional materials that can be potentially extracted from such crystals, computed the exfoliation energy and finally characterized the electronic properties of the extracted layers. These findings are an important contribution to the emerging field of non-vdW 2D materials since it provides for the first time a wide view of the materials space while previous studies have been focused only to specific families of materials, moreover the shear number of proposed two-dimensional systems is two orders of magnitude larger than previous works.
Overall, the paper is clearly written and well documented and substantiated.

I have some remarks on some specific aspects of the methodology and on the results:

1) The initial screening for the materials is done with what the authors call "universal exfoliation and cleavage potential" a pair-wise potential only dependent upon the interatomic distances with free parameters fitted for each element from the energies and forces in the bulk crystal database. In different crystal structures the same element and element pairs might be found in different chemical environments that could change the bond strenght in ways that are not fully captured by the interatomic distance alone. Fitting an element-wise potential thus necessarly results into an average value. To access the trasferability of the potential it would be useful to have even a rough estimate the width of the bond-strenght distribution across different chemical environments.

2) Always on the fitting of the potential, the author mention that they fitted the potential only for pairs represented in at least 20 cases but they don't mention whether the training set is balanced I.e. each pair is equally represented or some pairs are much more abundant than others. In this case, co sidering that the total loss function they minimize does not seem to be normalized with respect to the number of pair types it is likely that the loss function will be dominated by the more common pairs while, for which we can thus have a good representation, while less common pairs will have less reliable estimations.

3) Regarding the first principle calculations, both the initial bulk dataset and the calculations for the exfoliation energy have been carried out with the PBE functional. PBE is known to overestimate bond lengths and give a relatively poor description of weak bonds due to the unproper treatment of long range interactions. The authors should comment on the effects that such errors might have both in the fitting procedure and in the calculations of the binding energies.

4) The authors mention that they found 37208 layers starting from 20899 bulk structures however it is not clear if this number represent the total number of layers or it has been filtered for similar or identical structures. It would not be impossible for different bulk materials to produce the same 2d layers.

5) the authors found 2377 Two-dimensional materials, it would be useful to how many of such materials are unique to this database. In recent years in fact many 2d-materials databases have been produced, not only from exfoliation of vdw materials but also following bottom-up approaches and thus in principle contain structures without vdw nature.

6) I found it strange that materials with clearly layered structure and relatively moderate buckling like Bi and Sb are only found with the BONDDEL algorithm. Why HKLSEARCH is not able to identify them?

(Remarks on code availability)

Version 1:

Reviewer comments:

Reviewer #2

(Remarks to the Author)
My major comment was an inconsistency between the experimental results and the predictions: Only 6 out of 29 experimentally exfoliated non-vdW 2D materials satisfy the criteria of R>1.5, and even hematene (R=1.06) and silicene (R=1.13) do not satisfy the criteria.

To comply with this comment, the authors have added a paragraph: “Since we classify each surface identified by the XCP

approach as “cleavable”, these composition-conserving high-ratio materials can be considered “likely exfoliable” non-vdW 2D candidates. This does not imply that systems with smaller values of R cannot be exfoliated into non-vdW sheets, as demonstrated by a range of experiments discussed above. … Therefore, the cutoff at 1.5 is chosen. …”

It seems to be a reasonable discussion. However, it seems to be helpful if the authors would show the values of R for a few examples (hematite and silicene) in the Main Text rather than in the Supporting Information, because without the values of R, future readers may not understand the phrase with "as demonstrated by a range of experiments discussed above. "

Except for this point, the manuscript has been revised convincingly. The validity of 2D materials prediction has been supported by phonon calculations using UMLIP. The impact of the structural relaxation has been discussed, highlighting the difference of stability origin between vdW and non-vdW materials.

The comments from the reviewer #1 are very important for improving the quality of the stability and structural analyses. The revision also reflects those comments.

In this way, the manuscript has been improved significantly and I can recommend publication of this manuscript in Nature Communications.

(Remarks on code availability)
I have used the following software the authors suggested and confirmed that monolayer graphene can be identified from graphite.

https://xcp.hzdr.de

Reviewer #3

(Remarks to the Author)
The authors have effectively cleared my concerns in their response letter. In my opinion the paper can be published in it's present form.

(Remarks on code availability)

Open Access This Peer Review File is licensed under a Creative Commons Attribution 4.0 International License, which permits use, sharing, adaptation, distribution and reproduction in any medium or format, as long as you give appropriate credit to the original author(s) and the source, provide a link to the Creative Commons license, and indicate if changes were made.
In cases where reviewers are anonymous, credit should be given to 'Anonymous Referee' and the source.
The images or other third party material in this Peer Review File are included in the article’s Creative Commons license, unless indicated otherwise in a credit line to the material. If material is not included in the article’s Creative Commons license and your intended use is not permitted by statutory regulation or exceeds the permitted use, you will need to obtain permission directly from the copyright holder.

To view a copy of this license, visit https://creativecommons.org/licenses/by/4.0/

Response to reviewers for Barnowsky *et al*.

Reviewer #1 (Remarks to the Author):

Judging the current manuscript against its title, which emphasizes "exfoliation and cleavage of available bulk crystals," the central idea has been explored in previous studies (such as npj Computational Materials, 7(1), 147): 17062). Although the methods are different and while this manuscript does offer advantages over earlier works, these advances, in my view, are not sufficient to warrant publication in Nature Communications. By contrast, the alternative framing of the work—namely, the identification of a new class of two-dimensional materials via high-throughput theoretical methods—is considerably more compelling.

Authors:

We thank the reviewer for making us aware of these important previous studies and we agree with their suggestion to adapt the framing of our work in this respect. We have thus decided to sharpen the title explicitly towards non-vdW materials:

New title: "*Exfoliation and Cleavage of Non-van der Waals Materials from a Universal Potential*"

as well as to introduce a proper acknowledgement and discussion of the contributions to the field of crystal cleavage made by J. T. Paul *et al.*, npj Comput. Mater. **7**, 147 (2021) on page 3 of the manuscript:

"*A bonding-network topology approach for identifying cleavable surfaces by analyzing the breaking of a selected number of bonds has been introduced previously [41]. This method identifies potential cleavage planes from structural information alone, by examining the scaling of bonding networks with supercell size. The added value of our approach for cleavage lies in the additional quantification of bond strengths via the interatomic potential, which naturally provides a ranking of bonds and thereby indicates which bonds are most likely to be broken upon exfoliation. As a result, the method enables a direct estimation of relative surface formation energies without requiring explicit DFT calculations for each cleavage configuration.*"

In addition, as identified during further literature study, we also cite the new reference [21] (M. Ashton *et al.*, Phys. Rev. Lett. **118**, 106101 (2017)) in the introduction when discussing discovery approaches for layered 2D materials.

Reviewer #1 (Remarks to the Author):

I agree with the authors that the search for non–van der Waals (non-vdW) monolayers has so far relied heavily on trial-and-error approaches, and that a systematic theoretical investigation is indeed needed. However, the present manuscript does not report computational results required to support that the proposed 2,377 monolayers are stable and experimentally meaningful 2D materials.

Based on the information provided in the manuscript and the data availability webpage, MD simulations appear to have been performed for only a very limited number of systems (specifically those shown in Figure 4). In the absence of further clarification, this suggests that the stability analysis is restricted to six systems, which is insufficient to build confidence in a dataset exceeding 2,000 proposed non-vdW monolayers. I consider a robust stability analysis essential before proposing a new class of stable 2D materials. The authors themselves note the high surface reactivity of non-vdW systems (line 38), which further reinforces this concern. While neither phonon calculations nor convex-hull analysis can fully guarantee experimental stability, they are widely accepted as minimum baseline criteria in the field. Had such analyses been performed in a systematic manner—particularly in combination with the existence of the parent bulk phases—I would have regarded this work as a meaningful step forward.

Authors:

We appreciate these important remarks as well as the positive comments regarding the relevance of this work. The initial MD simulations were indeed only performed for the systems shown in Fig. 4 rendering a full stability classification necessary.

We note that during the refinement of the method within this revision process, the number of 2D candidates has slightly increased with further details described at the end of this response letter.

A description of the extensive stability analysis for all candidates performed during this revision is detailed below when responding to the “major issues” to be addressed.

Reviewer #1 (Remarks to the Author):

The authors largely base their claim regarding the existence of stable non–van der Waals (non-vdW) 2D materials on previously reported experimental cases. While such reports provide motivation for the present study, and while the results could potentially

help guide future experimental efforts, several critical issues must be considered. Given that most experimental studies in the literature (as collected in the Supporting Information) rely on liquid-phase exfoliation, two fundamental theoretical concerns must be addressed when proposing the resulting products as candidate 2D materials:

1. Monolayer versus few-layer stability. Are these materials stable as true monolayers, or do they only stabilize as few-layer thin films?

2. Chemical stability upon exfoliation. Since exfoliation from the bulk necessarily involves breaking strong covalent or ionic bonds, are the resulting layers intrinsically stable, or are they instead stabilized by passivation from ions or molecules present in the liquid environment?

As the present work is purely computational, I do not expect the authors to resolve these experimentally driven concerns directly. However, these considerations indicate that the existing experimental reports alone are insufficient to establish the existence of a stable class of non-vdW 2D materials, and that any theoretical claim must therefore be supported by a systematic stability analysis.

For these reasons, I cannot recommend publication of this manuscript in Nature Communications at this stage. I would, however, be willing to reconsider my recommendation if the following major issues are addressed:

Authors:

We thank the reviewer for this opportunity and address their issues below.

Reviewer #1 (Remarks to the Author):

1. Stability analysis: Phonon band calculations are essential, and convex-hull analysis is strongly encouraged, for the proposed 2D materials.

Authors:

After carefully considering and benchmarking different methods and options taking into account accuracy and feasibility, an investigation of the dynamical stability was performed for all 2531 candidates using high-throughput phonon analysis based on a machine learning interatomic potential (MLIP). Figure 3**f** (depicted below) now includes a statistic of maximum imaginary phonon frequencies over the dataset showcasing the large majority of systems to be vibrationally stable. The manuscript was extended accordingly on page 8:

“*A key criterion for predicting novel materials is their dynamical stability. To evaluate this, the phonon dispersion ω(q) for all DFT-relaxed structures are computed on a dense q-point mesh using the mace-mh-1 foundation model MLIP [73]. A material is considered dynamically stable if no imaginary frequencies appear anywhere in the first Brillouin zone. In practice, we quantify this by evaluating the maximum imaginary component. To account for numerical noise in the context of soft modes [74], a structure is classified as dynamically unstable only if this value exceeds 0.1 THz. This criterion is illustrated in Fig. 3**f**. The distribution of maximum imaginary frequencies exhibits a pronounced peak near zero, with a finite spread extending up to 0.1 THz. This peak corresponds to the dynamically stable majority, comprising 64.4 % of the systems. In contrast, unstable structures show significantly larger imaginary frequencies that are broadly distributed over several THz, resulting in a lower probability density. As such, instabilities do not preferentially occur near zero frequency. The observed fraction of stable systems lies between the near-100 % stability expected for vdW 2D materials, whose structures closely resemble their naturally layered bulk counterparts, and the 28.4 % stability reported for C2DB composed primarily of generated structures [70].*”

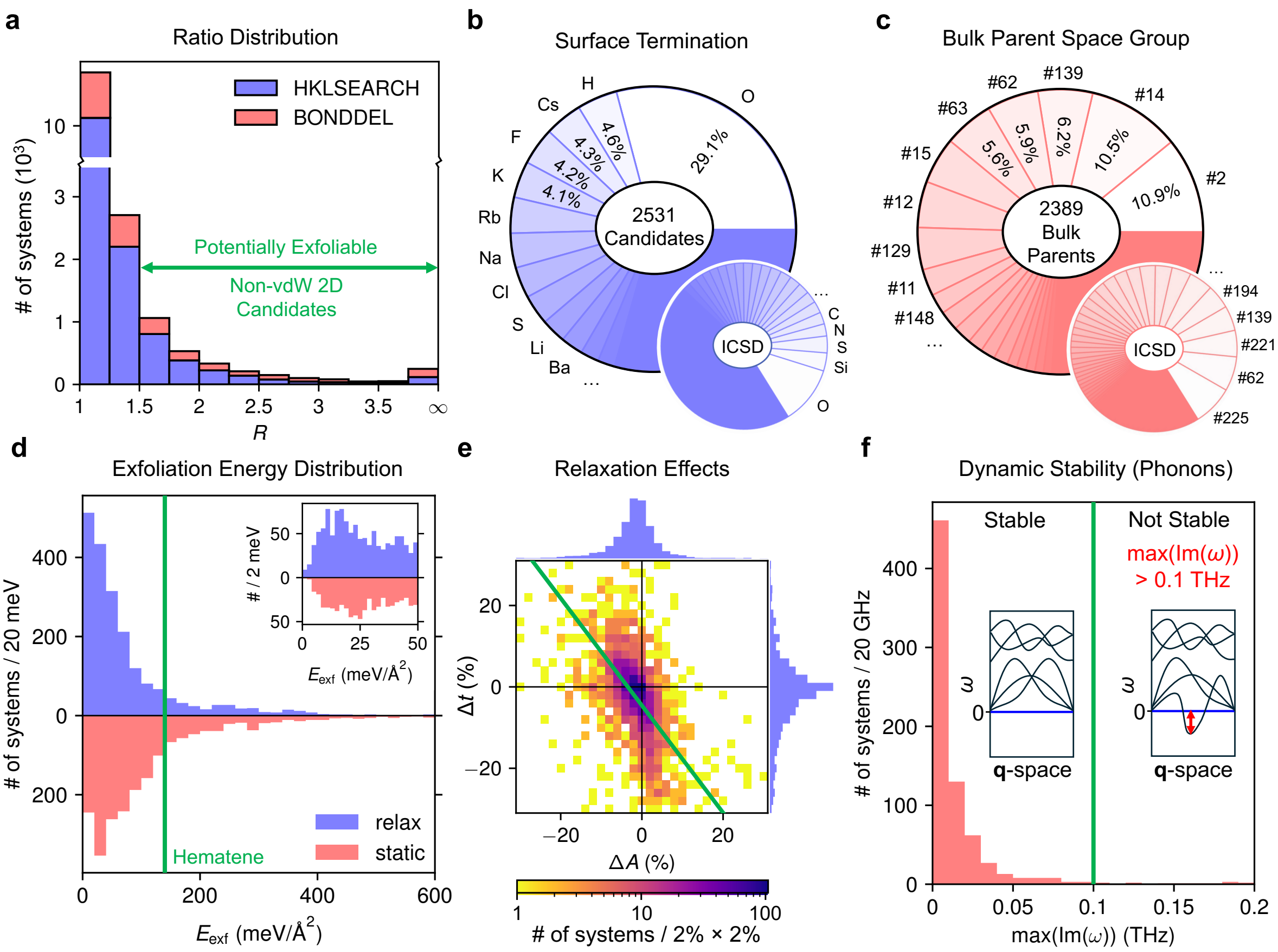

Revised Figure 3.

In addition, the Methods section was extended to include details about the employed approach on page 11-12:

"*Phonon calculations were performed using the mace-mh-1 MLIP [73]. This most recent MACE model was chosen as it was developed using cross-learning strategies and is therefore expected to perform well for structures that deviate from the typical PBE(+U) bulk training sets, such as the 2D sheets considered here. A second MLIP based on a similar philosophy, SevenNet-omni [87], was also tested and yields consistent trends. To ensure accurate forces, all DFT optimized structures were re-relaxed with the MLIP prior to the phonon calculations. This introduces a mismatch ϵ between the DFT-relaxed geometry and the MLIP ground state, which we quantify using the structure comparison implemented in AFLOW-XtalFinder [79]. Structures with $\epsilon < 0.1$ are considered equivalent; consequently, if the MLIP-relaxed structure satisfies this criterion, the MLIP is deemed sufficiently accurate for the corresponding candidate. This is the case for a total of 65.3 % of candidates that are then carried over to the statistics shown in Fig. 3**f**. A benchmark assessing the reliability of MLIP-predicted phonon dispersions for a representative set of non-vdW 2D candidates, compared with DFT ab initio results from Ref. [30], is provided in Supplementary Fig. 8. There a clear trend emerges: in four of the six cases, the MLIP-relaxed structure exhibits $\epsilon<0.1$ to the corresponding DFT-relaxed structure, consistent with the fraction observed across the full dataset. For these four materials, the MLIP phonon dispersions are in good agreement with the DFT results – particularly the low-frequency region that is most relevant for identifying dynamical instabilities. Phonon dispersions were computed within the finite-displacement framework as implemented in the atomic simulation environment (ASE) [88]. In-plane supercells were constructed dynamically to ensure a minimum separation of 40 Å between periodic images. The sampling of $\omega(q)$ was performed on a dense in-plane grid (2D-KPPRA = 25,000) to reliably capture the maximum imaginary frequency.*"

As some of the systems in Fig. 4 were not stable according to this more sophisticated analysis, the figure and description have been updated to include only dynamically stable systems.

The thermodynamic stability was investigated with respect to the suggested convex hull analysis. We first make use of the fact that the bulk parent materials of the 2D slabs are experimentally realized systems from the ICSD and hence are synthesizable. A layer-number dependent statistic of energies of the 2D systems relative to the convex hull is newly included in section II in the supporting information. The discussion has been added on page 6-7 of the main manuscript:

"*Another common measure of thermodynamic stability is the convex hull distance energy, ΔE_{hull}, i.e., the energetic distance of the 2D system to the convex hull of stability – a measure of energy per atom rather than per area [69]. We estimate this distance here with respect to the bulk parent structures taken from the AFLOW-ICSD dataset which have been realized experimentally. ΔE_{hull} can be related to the exfoliation energy*

E_{exf} via the atomic area density of the unrelaxed 2D sheet. While E_{exf} is the more natural quantity for 2D materials derived from bulk via exfoliation, ΔE_{hull} can be straightforwardly extended to stacks of N "monolayers" by the estimate, $\Delta E_{hull}(N) \approx \Delta E_{hull}(1)/N$. The resulting distributions of ΔE_{hull} for different N are shown in Supplementary Fig. 1. A commonly used criterion for thermodynamic stability is ΔE_{hull} < 100 meV/atom [70]. Based on this threshold, a fraction of 49.0 % of the candidates is thermodynamically stable in the monolayer limit, which increases rapidly to 70.0 % for bilayer stacks."

We also fully agree with the reviewer that further investigation into the microscopic details of the experimental process and the resulting non-vdW 2D structures is required and are thankful for their consideration that there is an inherent difficulty to make decisive claims in this regard from a purely theoretical perspective.

A full account on the open question regarding surface passivation and solvent interaction during the liquid-phase exfoliation experiments is beyond the scope of the present study. However, we agree that especially in the context of the discussion of the surface terminating species an additional remark in this respect is needed. We thus added the following text on page 5:

"*With around 30 %, most slabs are terminated by oxygen, usually in the −2 state, which is also the most common element among compounds in the AFLOW-ICSD, albeit with a smaller overall fraction. It is followed by H^+ (4.6 %), Cs^+ (4.3 %), F^-(4.2 %), and K^+ (4.1 %). However, this does not necessarily mean that experiments on non-vdW 2D materials will always be able to directly observe these terminations. Typical exfoliation techniques, especially LPE, work in a chemically complex environment that may lead to surface passivation by other species or functional groups.*"

Reviewer #1 (Remarks to the Author):

2. Structural analysis: The authors have access to structural data before and after monolayer relaxation, yet no quantitative analysis of bond deformation or reconstruction is presented. This analysis is crucial, as the novelty of these candidate 2D materials arises precisely from the non-intuitive stability of layers formed by breaking strong non-vdW bonds. The authors are in a unique position to comment on how the structures adjust upon exfoliation.

Authors:

Relaxations are indeed very important for non-vdW 2D materials. We have thus evaluated the structural change to the layers upon relaxation for the whole set based on area and thickness changes and incorporated this into the updated Fig. 3 in panel **e**. We add the following discussion on page 7-8:

"*The structural relaxations are substantial and follow a clear trend, as shown in Fig. 3**e** for the full dataset. The relative thickness change of the slab, Δt (with thickness defined as the vertical distance between the outermost atoms), is anti-correlated with the in-plane area change ΔA. These structural changes are in particular strong compared to vdW materials, which remain largely unchanged when extracted from their bulk parent structures. A set of examples demonstrating characteristic relaxation behavior of non-vdW 2D sheets is provided in Supplementary Fig. 2. A typical mechanism is the flattening of the broken coordination of surface ions, as exemplified by 2D (010) $MgSiO_3$ (ICSD #172736), which exhibits an area increase of 2.74 Å^2 (ΔA= 15 %) combined with a thickness reduction of 1.33 Å (Δt=−30 %). This can be attributed to the loss of out-of-plane bonding pulling surface ions towards the interior of the slab. In contrast, (111) K_2O (ICSD #641282) shows the opposite behavior, with an increase in thickness of 0.44 Å (Δt = 23 %) and a decrease in in-plane area of 3.24 Å^2 (ΔA=−18 %). Here, structural relaxation is driven by enhanced separation of K ions at opposite surfaces, leading to increased buckling. For more structurally complex materials, the same general trends persist, although a reduction to simple coordination arguments becomes increasingly difficult. For example, (001) SiO_2 (ICSD #171739) undergoes a long-range reconstruction, resulting in a large area increase of 53.20 A^2 (ΔA = 40 %) while preserving similar local atomic environments. Conversely, (001) OsP_4 (ICSD #647710) exhibits a near-complete reorganization of the P atoms around Os upon exfoliation, accompanied by a thickness increase of 0.87 Å (Δt = 23 %) and an area reduction of 9.01 Å^2 (ΔA=−18 %).*"

Reviewer #1 (Remarks to the Author):

Specific comments on the manuscript:

Abstract: The authors state, "we obtain 37,208 cleavable surfaces … from which we investigate 2,377 likely exfoliable ones." The criteria used to classify a surface as "cleavable" versus "exfoliable" must be explicitly defined.

Authors:

We acknowledge the necessity of a clearer definition. The sentence in the abstract is thus slightly adapted:

"Applying this approach, we obtain 44,030 cleavable surfaces and candidate non-vdW 2D materials from which we investigate – according to our criteria – 2,531 likely exfoliable ones using high-throughput density functional theory."

and we included the definition as soon as the necessary concepts regarding the exfoliability ratio are introduced on page 5:

"*Since we classify each surface identified by the XCP approach as "cleavable", these composition-conserving high-ratio materials can be considered "likely exfoliable" non-vdW 2D candidates.*"

Reviewer #1 (Remarks to the Author):

Line 76: The selection criteria for 2D material candidates are central to this work. Although Ref. [31] is cited, the relevant criteria should be clearly summarized in the present manuscript.

Authors:

The AFLOW-ICSD database referred to in the refence mentioned by the reviewer (now Ref. [32]) is a large repository of computational data for experimentally observed bulk crystal structures. These hence yield an ideal starting point for a computational investigation into the exfoliation of real materials. To clarify that this is an experimentally backed starting point, an appropriate remark has been added in the associated paragraph on page 2:

"*Application of the scheme to the AFLOW-ICSD database [32], a computational materials repository based on experimentally observed bulk crystal structures, leads to the identification of 2,531 novel exfoliable non-vdW 2D candidates with a wide variety in chemical composition, structure, and properties."*

We also mention this again explicitly at the beginning of the section "Predicting Novel Candidates" on page 5:

"*To identify new 2D materials, we focus on 3D bulk parent systems known from experiments. Hence, both approaches are applied to all unique bulk structures from the AFLOW ICSD dataset excluding layered materials (or assemblies of one- or zero-dimensional subunits held together by vdW forces) according to the standard descriptor introduced by Mounet et al. [22].*"

Reviewer #1 (Remarks to the Author):

Line 79: The authors refer to "properties" studied for the 2D candidates. Please specify for what fraction of the 2,377 monolayers MD and band-structure calculations were performed.

Authors:

As indicated above, the phonon analysis has now been performed for all systems within this revision. Band-structures have only been calculated for a small number of cases from which the examples shown in Fig. 4 were selected to demonstrate the principal behavior. We extended the description at the end of the introduction on page 2 accordingly:

"*High-throughput calculations for the whole set confirm preferable small exfoliation energies for the large majority of candidates and reveal that the main fraction is both dynamically and thermodynamically stable. The identified 2D materials class shows negligible overlap with existing databases and thus spans a largely unexplored chemical space. It includes interesting cases with hexagonal, square, and rectangular lattice symmetries exhibiting for instance metallic behavior or indirect-gap to direct-gap semiconductor transition upon exfoliation, as demonstrated for several selected examples.* "

Reviewer #1 (Remarks to the Author):

Line 158: Please clarify the meaning of "non-planar cleavage."

Authors:

We have clarified this with the following remark in the referenced sentence at the top right on page 3:

"*However, non-planar cleavage, i.e., when the cleavage surface is corrugated on an atomic scale rather than being a single planar cut, observed in materials such as*

tungsten trioxide (α-WO_3; ICSD #27961) [9], requires BONDDEL to be identified correctly."

Reviewer #1 (Remarks to the Author):

Line 182: Wulff constructions can provide insight into whether a material prefers to form thin films or three-dimensional clusters. Can the authors present or discuss Wulff-shape analysis for the systems studied?

Authors:

We have included the computed Wulff shapes for the six examples shown in Fig. 4 (shown below) as new panels **a**-**f** and added the following discussion on page 8:

"*Figure 4 showcases a selection of predicted dynamically stable non-vdW 2D systems starting from the Wulff shape of their bulk parents, over their relaxed slab geometry, to their electronic structure – underlining the variety of this rising class of materials. The Wulff shape is particularly interesting as for a flat appearance with dominant facets intuitively good cleavage properties can be expected. The examples are sorted vertically according to their lattice type, i.e., including novel hexagonal, square, and rectangular lattice systems.*"

And also discuss the special behavior of the BONDDEL algorithm on pages 8-9:

"*Sheets identified by BONDDEL are particularly intriguing, as they frequently exhibit non-planar surface terminations, such as the (001) $Ca_2Al_2SiO_7$ structure illustrated in Fig. 4**j**. In contrast to systems identified via HKLSEARCH, where the exfoliation plane and its symmetry-equivalent facets clearly dominate the Wulff shape as evident in Figs. 4**a**–**b** and **d**–**e**, this feature is much less pronounced for systems with corrugated surfaces (Fig. 4**c**). The reason is that such cuts cannot be represented by planar (hkl) facets within the XCP surface energy formalism.*"

The updated Figure 4 is shown below:

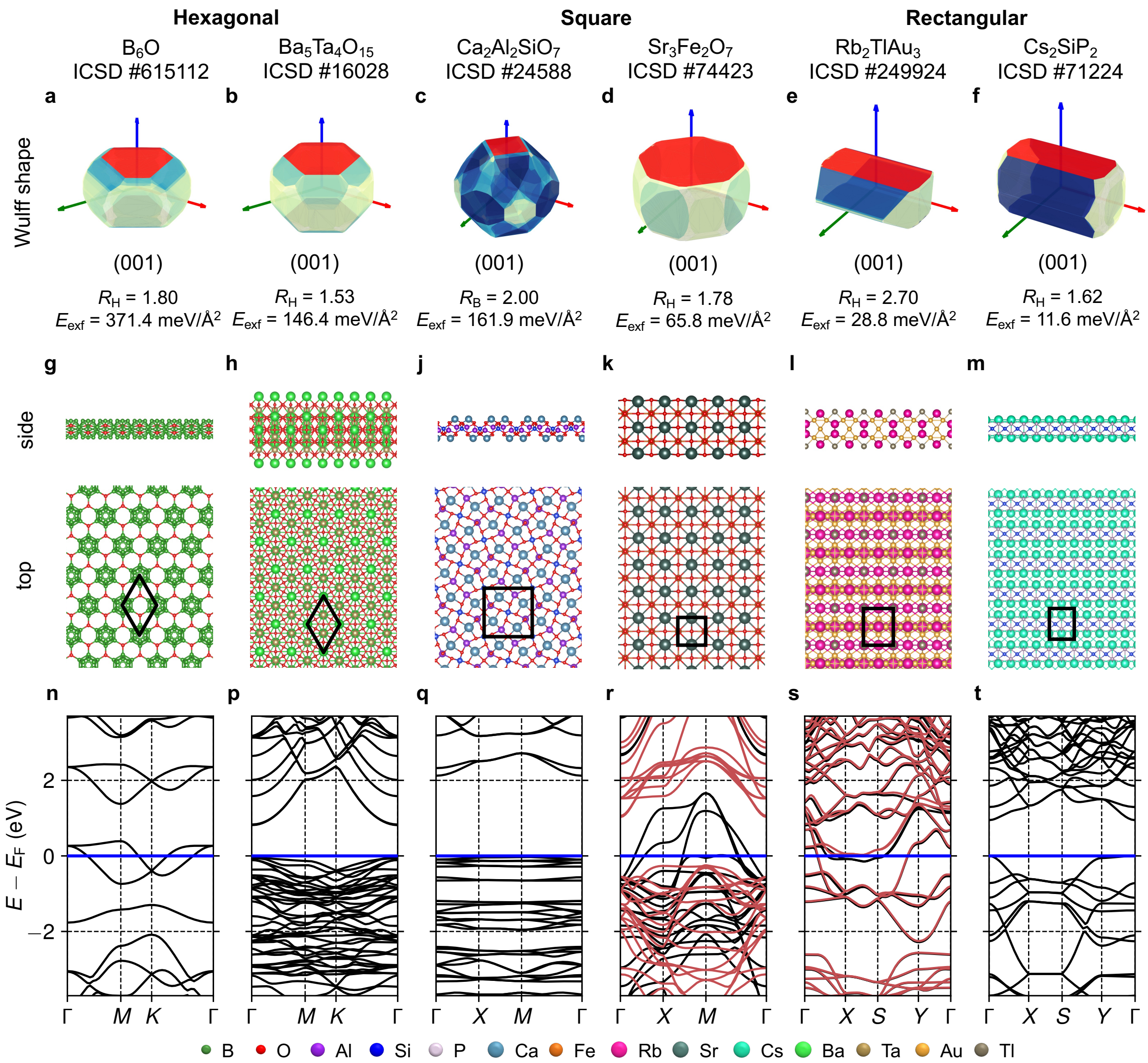

Revised Figure 4.

Reviewer #1 (Remarks to the Author):

Line 270: The authors state that exfoliation energies are calculated only for systems "whose composition is conserved." Please clarify how this criterion is applied in practice. Specifically, how many (or what fraction) of the 2,377 proposed exfoliable monolayers preserve their composition and are therefore carried forward to the AFLOW/VASP calculations? Alternatively, does the number 2,377 already correspond to this subset? Please also clarify whether lattice relaxations are performed exclusively for these systems, and how many materials are excluded based on this criterion.

Line 273: What is the origin of the 16,397 systems mentioned here? Why is this number smaller than the total number of 2D systems discussed earlier?

Authors:

All these numbers refer to composition-conserving systems. We perform DFT calculations for 2,531 out of 17,897 systems based on the criterion R > 1.5. To make this clearer, we have improved the formulations on page 5:

"*For further analysis, especially for computing exfoliation energies E_{exf} [66], only systems whose composition is conserved upon exfoliation are considered (excluding, e.g., MXenes). The compositions of both the bulk and 2D systems are compared for each candidate individually.*
*The distribution of these 17,897 composition-conserving systems as a function of the exfoliability ratio R is shown in Fig. 3**a**. As expected, the majority of systems exhibits low ratios close to unity, indicating that they are very isotropic and not readily exfoliable. However, a significant number of several thousand systems show higher ratios, suggesting that they are promising candidates for non vdW 2D materials. Consequently, out of these, we decided to perform a detailed high-throughput analysis including structural relaxations on the 2,531 unique composition conserving systems with R > 1.5.*"

Reviewer #1 (Remarks to the Author):

Line 282: The surface termination analysis is interesting; however, given that liquid-phase exfoliation occurs in a chemically active and uncontrolled environment, do the authors expect that experimentally realized thin films may be stabilized by surface passivation?

Authors:

This is indeed expected. As briefly mentioned above, we have modified the text on page 5 accordingly:

"*With around 30 %, most slabs are terminated by oxygen, usually in the −2 state, which is also the most common element among compounds in the AFLOW-ICSD, albeit with a smaller overall fraction. It is followed by H^+ (4.6 %), Cs^+ (4.3 %), F^-(4.2 %), and K^+ (4.1 %). However, this does not necessarily mean that experiments on non-vdW 2D materials will always be able to directly observe these terminations. Typical exfoliation techniques, especially LPE, work in a chemically complex environment that may lead to surface passivation by other species or functional groups.*"

Reviewer #1 (Remarks to the Author):

Line 359: How were the Hubbard U values chosen for each element and compound? Were these U values also used during structural relaxations? While such information may exist in previous AFLOW publications, I recommend that sufficient computational details be provided to make the present manuscript self-contained.

Authors:

We agree that the manuscript should be self-contained and thus additional information has been added to the methods section on page 11 as well as the new section VII. in the SI has been established including a summary of the AFLOW standard settings relevant for this work.

"*All DFT calculations are performed using the Vienna Ab-Initio Simulation Package (VASP) [80–84] with the PBE(+U) functional [33–36], employing the AFLOW software [85] and its standard settings [86] (for details see Section VII in the Supplementary Information) to ensure compatibility with the AFLOW database. Reciprocal space integration grids for relaxations were determined using 2D-KPPRA = 400 while the subsequent static calculation, used to evaluate the total energy and DOS, employs 2D-KPPRA = 2,500. The band-structure calculations shown in Fig. 4 were performed based on the static geometry and charge density. The on-site U values listed in Supplementary Tables 2 and 3 are employed during geometry optimization as well as static and band structure calculations.*"

Reviewer #2 (Remarks to the Author):

Barnowsky et al. have explored non-van der Waals (non-vdW) 2D materials by computational exfoliation and cleavage of known crystals. They constructed a model potential (XCP) that is a sum of Morse and Yukawa potentials, which computes the bond strength with small computational costs. They also introduced BONDDEL and HKLSEARCH schemes, which predict known non-vdW 2D materials such as alpha-WO3 and silicene, respectively, and defined the exfoliability ratio R in Eq. (2). Using these approaches, they proposed that 2377 exfoliable monolayers from non-vdW materials. The statistical analysis and electronic structure calculations have also been performed. finding It remains an open question how to predict non-vdW 2D materials, so the idea proposed in the present manuscript is interesting. However, I do not agree that this approach successfully identifies known 2D materials and accurately predicts likely exfoliable ones.

Authors:

We thank the reviewer for their encouraging remarks about the relevance of the proposed concepts. Their concerns will be addressed below.

We note that during the refinement of the method within this revision process, the number of 2D candidates has slightly increased with further details described at the end of this response letter.

Reviewer #2 (Remarks to the Author):

Major comments
At line 280, the authors state that "we decided to perform a detailed high-throughput analysis on the 2377 systems with R>1.5". On the other hand, only 6 out of 29 experimentally exfoliated non-vdW 2D materials satisfy the criteria of R>1.5, and even hematene (R=1.06) and silicene (R=1.13) do not satisfy the criteria, which are shown in Supporting Information. There is a clear inconsistency between the experimental results and the predictions, indicating an apparent contradiction in this manuscript.

Authors:

The contradiction can actually be resolved. The experimentally realized materials like hematene are not necessarily more easily exfoliable then most others, they just happen to be experimentally tested first mostly by "trial and error" choice. If one – without knowing the ratio distribution in Fig. 3**a** (shown below) right away – would randomly pick a material (and test it for exfoliability), one would simply due to probability most likely choose one with a small ratio as most of the bulk systems are in this regime. As such, that in the listing of the previously experimentally realized non-vdW 2D systems there are only a few with high ratios is not surprising and merely reflects the ratio distribution over all bulk systems.

On the other hand, from the computational point of view, our model of course only has a finite fitting accuracy due to *e.g.* numerical effects and the two-body term approximation. Hence, the smaller the ratio becomes, the more small contributions to the bonding strength in different directions will be important which the model can capture only to certain extent. In order to avoid these issues, we chose systems with R>1.5 with a clear bond strength anisotropy as a conservative estimate where we can clearly expect the XCP model to yield reliable predictions. This already provides the outlined several thousand candidates and

an investigation of systems with smaller ratios might follow in the future. The fact that we have already identified a vast range of materials that are more easily exfoliable (according to our ratio values but also exfoliation energies) than those produced in previous experiments underscores the potential of the new non-vdW 2D materials class. We added the following discussion on page 5:

"*Since we classify each surface identified by the XCP approach as "cleavable", these composition-conserving high-ratio materials can be considered "likely exfoliable" non-vdW 2D candidates. This does not imply that systems with smaller values of R cannot be exfoliated into non-vdW sheets, as demonstrated by a range of experiments discussed above. However, due to the finite accuracy of the XCP model to estimate relative bond strengths in different directions, the predictive power of the present model is expected to increase for larger values of R. Therefore, the cutoff at 1.5 is chosen conservatively to ensure that the candidates identified here are highly likely to be practically exfoliable – potentially even with less effort than many of the previously reported experimental examples.*"

The reason for the increase of the number of 2D candidates from 2,377 to 2,531 in this revision is addressed under "Additional Changes" at the end of this document.

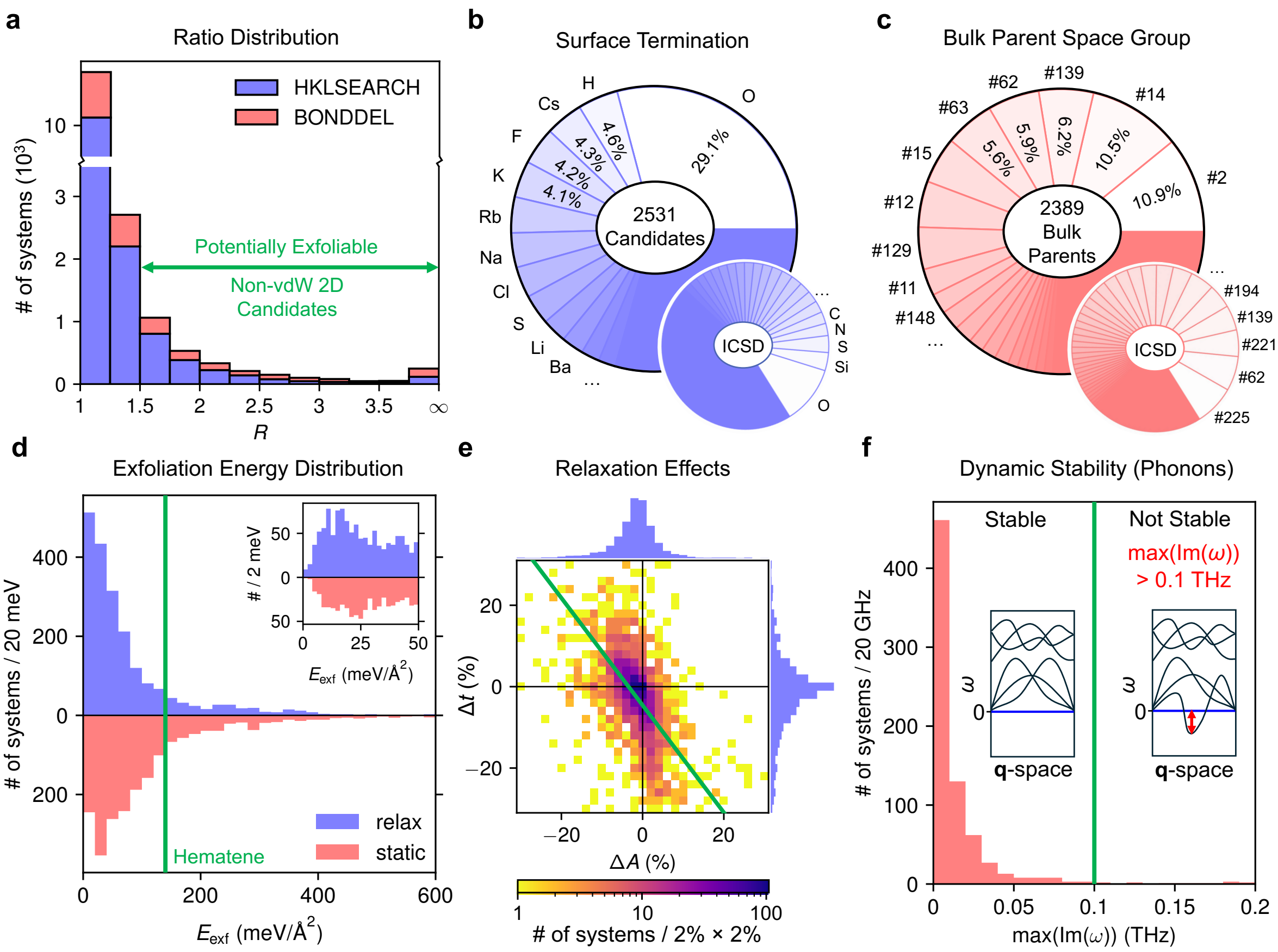

Revised Figure 3.

Reviewer #2 (Remarks to the Author):

In my understanding, the surface energy, corresponding to R_H estimated by the HKLSEARCH, may not be an adequate descriptor for predicting non-vdW 2D materials. As mentioned in lines 333-337, the final structures are determined by the surface energy. However, the growth process of non-vdW 2D materials may not be solely determined by the surface energy.

Authors:

We thank the reviewer for this important remark. It is indeed not directly possible to claim which materials are growable just by investigating their surface energies and we did not intend to make this claim. For this reason, the primary focus of the manuscript is on exfoliability where surface energy should play a decisive role. However, the surface energy can also determine the final shape of grown flakes and particles especially related to the Wulff construction providing the equilibrium shape of an unperturbed crystal. We can hence make predictions in this regard using the XCP model. To clarify this, the paragraph on page 5 has been updated accordingly:

"*In addition, the XCP approach can also be valuable in the context of certain growth processes. While kinetics plays a decisive role during growth, often the final shapes of particles and platelets are determined by the minimization of surface energy as outlined by the Wulff construction, which can thus be captured by XCP.*"

Reviewer #2 (Remarks to the Author):

Minor comments
Lines 62-64. The discussion of Refs. [24-27] is rather superficial. A more careful discussion is needed to clarify the differences from the present work.

Authors:

We acknowledge the need for a more detailed discussion of the mentioned references and have thus expanded the relevant paragraph on page 1 to 2 (Note the increase of the reference numbers by one due to a new reference inserted earlier in the manuscript):

"*Other approaches are limited to computationally expensive methods such as the calculation of elastic constants [25] or explicit electronic structure evaluations for each surface. These are often preceded by simple pre-screening schemes based on packing*

ratios [26] or bond density [27], which are used to identify potentially relevant bulk facets. In parallel, there have been conceptual approaches aimed at distinguishing non-vdW from conventional vdW materials [28], as well as studies focused on specific materials classes such as hematene-like structural prototypes [29, 30] and MXenes [31]."

Reviewer #2 (Remarks to the Author):

Lines 396-398. The six systems in Fig. 4 were found to be dynamically stable through density functional MD simulations. Does this guarantee no unstable phonon mode in the Brillouin zone? Why were phonon dispersions not calculated?

Authors:

This is a very important remark as we found that unstable phonon modes cannot be fully excluded by an MD simulation especially during the relatively short simulation time in the original investigation. We address this point in this revision after carefully considering and benchmarking different methods and options by systematically investigating the dynamical stability using an MLIP-based high-throughput phonon analysis. Figure 3 (shown above) was adjusted with the new panel **f** to include a statistic of maximum imaginary phonon frequencies over the dataset. As some of the systems in Fig. 4 were not stable according to this more sophisticated criterion, the Figure and description has been updated to include only dynamically stable systems. The manuscript was extended with respect to the phonon analysis accordingly on page 8:

"*A key criterion for predicting novel materials is their dynamical stability. To evaluate this, the phonon dispersion ω(q) for all DFT-relaxed structures are computed on a dense q-point mesh using the mace-mh-1 foundation model MLIP [73]. A material is considered dynamically stable if no imaginary frequencies appear anywhere in the first Brillouin zone. In practice, we quantify this by evaluating the maximum imaginary component. To account for numerical noise in the context of soft modes [74], a structure is classified as dynamically unstable only if this value exceeds 0.1 THz. This criterion is illustrated in Fig. 3**f**. The distribution of maximum imaginary frequencies exhibits a pronounced peak near zero, with a finite spread extending up to 0.1 THz. This peak corresponds to the dynamically stable majority, comprising 64.4 % of the systems. In contrast, unstable structures show significantly larger imaginary frequencies that are broadly distributed over several THz, resulting in a lower probability density. As such, instabilities do not preferentially occur near zero frequency. The observed fraction of stable systems lies between the near-100 % stability expected for vdW 2D materials,*

whose structures closely resemble their naturally layered bulk counterparts, and the 28.4 stability reported for C2DB composed primarily of generated structures [70]."

In addition, the Methods section was extended to include details about the employed approach on page 11:

"*Phonon calculations were performed using the mace-mh-1 MLIP [73]. This most recent MACE model was chosen as it was developed using cross-learning strategies and is therefore expected to perform well for structures that deviate from the typical PBE(+U) bulk training sets, such as the 2D sheets considered here. A second MLIP based on a similar philosophy, SevenNet-omni [87], was also tested and yields consistent trends. To ensure accurate forces, all DFT optimized structures were re-relaxed with the MLIP prior to the phonon calculations. This introduces a mismatch ϵ between the DFT-relaxed geometry and the MLIP ground state, which we quantify using the structure comparison implemented in AFLOW-XtalFinder [79]. Structures with ϵ < 0.1 are considered equivalent; consequently, if the MLIP-relaxed structure satisfies this criterion, the MLIP is deemed sufficiently accurate for the corresponding candidate. This is the case for a total of 64.9 % of candidates that are then carried over to the statistics shown in Fig. 3**f**. A benchmark assessing the reliability of MLIP-predicted phonon dispersions for a representative set of non-vdW 2D candidates, compared with DFT ab initio results from Ref. [30], is pro vided in Supplementary Fig. 8. There a clear trend emerges: in four of the six cases the MLIP-relaxed structure exhibits ϵ<0.1 to the corresponding DFT-relaxed structure, consistent with the fraction observed across the full dataset. For these four materials, the MLIP phonon dispersions are in good agreement with the DFT results, particularly the low-frequency region that is most relevant for identifying dynamical instabilities. Phonon dispersions were computed within the finite-displacement framework as implemented in the atomic simulation environment (ASE) [88]. In-plane supercells were constructed dynamically to ensure a minimum separation of 40 Å between periodic images. The sampling of ω(q) was performed on a dense in-plane grid (2D-KPPRA = 25,000) to reliably capture the maximum imaginary frequency.*"

Reviewer #2 (Remarks on code availability):

Example files for FINDSLAB_POTDATA are not provided, and I could not run the code.

Authors:

In the initial software release, the potential data was not published with the code as we have considered it as data and included it in the data publication instead. To make it easier to run the code, we have now also included a copy of the potential data with the code under the mentioned link as well as created an

interactive website to run the predictions on crystal structures provided by the user. The section “Code Availability” on page 12 was updated accordingly:

“*The methods developed in this work can be employed interactively for any input crystal structure at https://xcp.hzdr.de. In addition, the software implementing the approaches is openly available on RODARE at https://doi.org/10.14278/rodare.4180 [90].*”

Reviewer #3 (Remarks to the Author):

In this work the authors propose a general methodology to discover new two-dimensional materials starting from non-Van der Waals bonded bulk crystals and then they apply such methodology to a large database of pre-processed crystal structures (i.e. structure coming from an experimental database but previously optimized with density functional theory). In their quest the authors successfully identified a large number of possible two-dimensional materials that can be potentially extracted from such crystals, computed the exfoliation energy and finally characterized the electronic properties of the extracted layers. These findings are an important contribution to the emerging field of non-vdW 2D materials since it provides for the first time a wide view of the materials space while previous studies have been focused only to specific families of materials, moreover the shear number of proposed two-dimensional systems is two orders of magnitude larger than previous works.

Overall, the paper is clearly written and well documented and substantiated.

Authors:

We sincerely appreciate the reviewer’s supportive comments regarding the quality and relevance of our work.

We note that during the refinement of the method within this revision process, the number of 2D candidates has slightly increased with further details described at the end of this response letter.

Reviewer #3 (Remarks to the Author):

I have some remarks on some specific aspects of the methodology and on the results:

Authors:

These comments will be addressed one-by-one below.

Reviewer #3 (Remarks to the Author):

1) The initial screening for the materials is done with what the authors call "universal exfoliation and cleavage potential" a pair-wise potential only dependent upon the interatomic distances with free parameters fitted for each element from the energies and forces in the bulk crystal database. In different crystal structures the same element and element pairs might be found in different chemical environments that could change the bond strength in ways that are not fully captured by the interatomic distance alone. Fitting an element-wise potential thus necessarily results into an average value. To access the transferability of the potential it would be useful to have even a rough estimate the width of the bond-strength distribution across different chemical environments.

Authors:

This remark touches a valid limitation of the potential as the chemical environment has an influence which is averaged in the current approach. However, the potential is not intended to be a general interatomic potential for applications like molecular dynamics, but it should mainly determine the bond strengths in an accurately optimized structure. As such, a structure implicitly captures much of the local chemistry via different characteristic bond lengths which is transferred to the length dependent potential fit. We have addressed this in detail as mentioned in the main text on page 3:

"*Further details as well as a verification of the developed interatomic potential also discussing the influence of different chemical environments are included in the Methods section.*"

and added a discussion to the "Potential Validation" part of the Methods section on page 10 as well as included a quantitative analysis in section VI. in the SI.

"*Constructing the potential energy such that it solely depends on atomic species and pairwise interatomic distances allows the model to describe interactions across the full periodic table with a comparatively small number of parameters. This is in contrast to approaches that explicitly incorporate oxidation states or other local environment descriptors. This simplicity, however, also limits its accuracy, as bond strengths are influenced by the local chemical environment. At the same time, many of these local*

effects are implicitly encoded in a quantity the potential depends on: the interatomic distance. This is because the local environment affects not only bond strength but also the characteristic bond length. Provided that the underlying structure is obtained from an accurate method, these geometric features already reflect the local chemical environment to large extent. As a result, a potential that depends only on species and distance can still distinguish between different chemical environments. This is exemplified in Supplementary Fig. 7 for the O–Fe interaction, where the common Fe oxidation states +2 and +3 separate in energy rather well based solely on their characteristic bond lengths [76]. Consequently, the model is well suited for evaluating bond strengths in given structures, while it is not intended for structure optimization or molecular dynamics, where the lack of explicit environment dependence would tend to homogenize bond lengths across different chemical environments."

Reviewer #3 (Remarks to the Author):

2) Always on the fitting of the potential, the author mention that they fitted the potential only for pairs represented in at least 20 cases but they don't mention whether the training set is balanced I.e. each pair is equally represented or some pairs are much more abundant than others. In this case, co sidering that the total loss function they minimize does not seem to be normalized with respect to the number of pair types it is likely that the loss function will be dominated by the more common pairs while, for which we can thus have a good representation, while less common pairs will have less reliable estimations.

Authors:

We thank the reviewer for this comment. We have not additionally balanced our training data against the number of cases for each bond type. This was an intentional choice as the model was trained on a dataset (AFLOW-ICSD) which roughly corresponds to the distribution of species and bond pairs present in real materials. Hence the current potential performs best for most materials it is applied to, while making tradeoffs for less common chemistries. This is now addressed in the Methods section on page 10 and in an analysis quantifying the representation bias shown in section V. in the SI.

"*The loss function and training set are not additionally balanced with respect to the number of occurrences of specific species combinations. As shown in Supplementary Fig. 5, the distribution of bond types is biased toward common species, meaning that frequently occurring combinations contribute more strongly to the fit. As a consequence, these bond types are represented with higher accuracy in the resulting*

potential. Given that the species distribution in the AFLOW-ICSD dataset can be considered representative of real materials, this imbalance aligns the model with physically relevant configurations. In comparison to a balanced approach, it therefore improves accuracy for previously unseen materials whose compositions follow the same underlying distribution as the training data, while less common species combinations are correspondingly less accurately described."

Reviewer #3 (Remarks to the Author):

3) Regarding the first principle calculations, both the initial bulk dataset and the calculations for the exfoliation energy have been carried out with the PBE functional. PBE is known to overestimate bond lengths and give a relatively poor description of weak bonds due to the unproper treatment of long range interactions. The authors should comment on the effects that such errors might have both in the fitting procedure and in the calculations of the binding energies.

Authors:

We thank the reviewer for this remark. PBE does systematically give too long bonds and might underestimate interaction strength. However, we do not expect a significant impact on the predicted candidates. For the potential fitting, the model is consistently trained on and applied to PBE structures and hence the particularities of PBE will be largely cancelled. To more clearly state the limitation of the potential fit due to the use of PBE reference values, we address this in more detail on page 3 of the manuscript:

"*We note that the AFLOW-ICSD reference values used for fitting are obtained from PBE(+U) DFT calculations [33–36]. Consequently, the resulting XCP inherits the systematic characteristics of this level of theory. In particular, PBE is known to underestimate interactions with a significant dispersive contribution, implying that the XCP will likewise tend to underestimate bonding energies in such cases. Due to the consistent training and application of the model, these effects are, however, expected to effectively cancel when predicting novel 2D candidates.*"

This of course also applies to energies computed using this level of theory, we hence state the usage of PBE directly on page 5 when exfoliation energies are first introduced:

"*Fig. 3**d** shows the distribution of computed PBE(+U) DFT exfoliation energies obtained by comparison with the corresponding bulk AFLOW entries.*"

Reviewer #3 (Remarks to the Author):

4) The authors mention that they found 37208 layers starting from 20899 bulk structures however it is not clear if this number represent the total number of layers or it has been filtered for similar or identical structures. It would not be impossible for different bulk materials to produce the same 2d layers.

Authors:

We thank the reviewer for this important remark. We deem such a uniqueness check not directly required for all cleavable planes as if two bulks lead to the same surface structure, these can still be considered as two separate physical situations. However, the final set of materials deemed exfoliable 2D candidates clearly needs to undergo such an investigation. In fact, out of 2,552 materials with R>1.5 2,531 are unique according to the criterion outlined below. The analysis in this revision focuses hence only on the unique entries. We accordingly adapted the text on page 5:

"Consequently, out of these, we decided to perform a detailed high-throughput analysis including structural relaxations on the 2,531 unique composition conserving systems with R > 1.5."

The uniqueness check is described on page 11 in the Methods section:

*"**2D Material Comparisons**. Uniqueness within the set of 2,531 exfoliable candidates, as well as overlap with existing 2D materials databases, is assessed using a hashing scheme that assigns each material a unique string of the form <stoichiometry>_xcp_<XCP total energy>. Here the energy is expressed as a value in eV/atom up to two significant figures. While this does not constitute a structural comparison at the level of more advanced tools such as AFLOW-XtalFinder [79], the combination of stoichiometry and total energy makes accidental collisions between distinct 2D materials highly unlikely. This approach is applied to both unrelaxed and relaxed structures: the former to avoid redundant DFT calculations on equivalent candidates, and the latter to enable direct comparison with ground-state structures reported in external materials databases."*

Reviewer #3 (Remarks to the Author):

5) the authors found 2377 Two-dimensional materials, it would be useful to how many of such materials are unique to this database. In recent years in fact many 2d-materials

databases have been produced, not only from exfoliation of vdw materials but also following bottom-up approaches and thus in principle contain structures without vdw nature.

Authors:

To address this remark, we computed the overlap between the predicted materials and the 2D databases MC2D, 2DMatPedia, and C2DB. The analysis yielded negligible overlap to all of them. This shows the orthogonality of the non-vdW 2D class to conventional 2D materials and strengthens the manuscript significantly. The following text was added on page 8:

"*As it cannot be excluded that some of the 2D candidates predicted in this work have previously been found in the context of other high-throughput 2D materials studies, we investigate the overlap with other databases in detail. Significant overlap with vdW-materials-based datasets such as the Materials Cloud 2D Database (MC2D) [71] and 2DMatPedia [72] is unlikely, since layered compounds were excluded from the input structures to which the descriptor was applied. There might be greater potential for overlap with the Computational 2D Materials Database (C2DB), which is largely based on bottom-up generation of 2D candidates [70]. However, in practice we observe zero overlap with the MC2D, only three materials are found by the present work and 2DMatPedia as well as two duplicate materials with respect to the C2DB are identified. Comparisons are performed using a stoichiometry and total energy based scheme outlined in the Methods section. These results indicate that non-vdW 2D materials form a new class orthogonal to conventional 2D materials, thereby substantially expanding the accessible design space for 2D compounds.*"

As well as the necessary information in the Methods section already mentioned above:

"***2D Material Comparisons**. Uniqueness within the set of 2,531 exfoliable candidates, as well as overlap with existing 2D materials databases, is assessed using a hashing scheme that assigns each material a unique string of the form <stoichiometry>_xcp_<XCP total energy>. Here the energy is expressed as a value in eV/atom up to two significant figures. While this does not constitute a structural comparison at the level of more advanced tools such as AFLOW-XtalFinder [79], the combination of stoichiometry and total energy makes accidental collisions between distinct 2D materials highly unlikely. This approach is applied to both unrelaxed and relaxed structures: the former to avoid redundant DFT calculations on equivalent candidates, and the latter to enable direct comparison with ground-state structures reported in external materials databases.*"

Reviewer #3 (Remarks to the Author):

6) I found it strange that materials with clearly layered structure and relatively moderate buckling like Bi and Sb are only found with the BONDDEL algorithm. Why HKLSEARCH is not able to identify them?

Authors:

This behavior of HKLSEARCH arises due to the much lower bond density of these materials across their {012} planes in contrast to {001} and the comparatively strong interlayer interaction. While in a typical covalent-radii-based atomic structure visualization the material appears layered, the Bi-Bi bonds between the layers have still around 80% of the strength of their intralayer counterparts. Hence the classification of these materials is non-vdW.

This leads to XCP-HKLSEARCH determining the lowest energy surfaces as {012}. These results show the limitations of a purely static approximation as well as highlight three important caveats already incorporated into the methodology:

1. Energies are very important for meaningful cleavage predictions. Approaches purely based on bond density or bond count will necessarily have a much lower accuracy.

2. The model is increasingly descriptive for high ratios. Bi(012) exhibits a ratio of 1.07 in the HKLSEARCH approach and hence would not nearly be classified as exfoliable according to the employed R>1.5 cut-off.

3. BONDDEL is important even for planar cuts and the two approaches do not necessarily agree.

While we do not discuss the failure modes for each individual example, there have been a range of changes made in this revision which will help to clarify these points including the following additions on page 3 and 5:

"*A bonding-network topology approach for identifying cleavable surfaces by analyzing the breaking of a selected number of bonds has been introduced previously [41]. This method identifies potential cleavage planes from structural information alone, by examining the scaling of bonding networks with supercell size. The added value of our approach for cleavage lies in the additional quantification of bond strengths via the interatomic potential, which naturally provides a ranking of bonds and thereby indicates which bonds are most likely to be broken upon exfoliation. As a result, the*

method enables a direct estimation of relative surface formation energies without requiring explicit DFT calculations for each cleavage configuration."

"*Therefore, the cutoff at 1.5 is chosen conservatively to ensure that the candidates identified here are highly likely to be practically exfoliable – potentially even with less effort than many of the previously reported experimental examples.*"

Additional changes

Due to a subtle programming error in the employed FINDSLAB software, a small range of structures had not been included in the initial analysis. In detail, the software failed without notice if the separation between parallel equivalent (hkl) planes became too small so a sweep along the plane normal as indicated in Fig. 2**c** and **d** was no longer possible within the given numerical accuracy. This only occurs for high-index planes and does not have any effect on the already predicted 2D materials. However it caused several bulk structures to not yield results. This has since been corrected and the code is updated accordingly in the code publication. The full analysis presented in this revision has been performed including these additional systems with extensive testing. Due to this, the overall numbers have slightly increased throughout the manuscript as well as minor changes to the statistics can be observed. The changes in the manuscript text related to this have not been marked in blue to not distract from the parts where the reviewer's comments are addressed.

We have become aware of the following article and added it as a second relevant reference for Si exfoliation (Wang *et al.*, J. Phys. Chem. Lett. **11**, 8668–8674 (2020)) as Ref. [43].

Response to reviewers for Barnowsky *et al*.

Reviewer #2 (Remarks to the Author):

My major comment was an inconsistency between the experimental results and the predictions: Only 6 out of 29 experimentally exfoliated non-vdW 2D materials satisfy the criteria of R>1.5, and even hematene (R=1.06) and silicene (R=1.13) do not satisfy the criteria.

To comply with this comment, the authors have added a paragraph: "Since we classify each surface identified by the XCP approach as "cleavable", these composition-conserving high-ratio materials can be considered "likely exfoliable" non-vdW 2D candidates. This does not imply that systems with smaller values of R cannot be exfoliated into non-vdW sheets, as demonstrated by a range of experiments discussed above. ... Therefore, the cutoff at 1.5 is chosen. ..."

It seems to be a reasonable discussion. However, it seems to be helpful if the authors would show the values of R for a few examples (hematite and silicene) in the Main Text rather than in the Supporting Information, because without the values of R, future readers may not understand the phrase with "as demonstrated by a range of experiments discussed above. "

Except for this point, the manuscript has been revised convincingly. The validity of 2D materials prediction has been supported by phonon calculations using UMLIP. The impact of the structural relaxation has been discussed, highlighting the difference of stability origin between vdW and non-vdW materials.

The comments from the reviewer #1 are very important for improving the quality of the stability and structural analyses. The revision also reflects those comments.

In this way, the manuscript has been improved significantly and I can recommend publication of this manuscript in Nature Communications.

Authors:

Thank you very much for these positive remarks. To address the remaining comment we have added the ratio values for hematene and silicene to the manuscript text.

"*This does not imply that systems with smaller values of R cannot be exfoliated into non-vdW sheets, as demonstrated by a range of experiments discussed above, e.g., R = 1.06 for hematene and R = 1.13 for silicene.*"

Reviewer #2 (Remarks on code availability):

I have used the following software the authors suggested and confirmed that monolayer graphene can be identified from graphite.

https://xcp.hzdr.de

Authors:

We are happy that the reviewer as worked with our online tool and can confirm its reliability.

Reviewer #3 (Remarks to the Author):

The authors have effectively cleared my concerns in their response letter. In my opinion the paper can be published in it's present form.

Authors:

We thank the reviewer for their positive comment.
